# Characterizing the transitioning epidemiology of herpes simplex virus type 1 in the USA: model-based predictions

**DOI:** 10.1186/s12916-019-1285-x

**Published:** 2019-03-11

**Authors:** Houssein H. Ayoub, Hiam Chemaitelly, Laith J. Abu-Raddad

**Affiliations:** 1 Department of Mathematics, Statistics, and Physics, Qatar University, P.O. Box 2713, Doha, Qatar; 2 Infectious Disease Epidemiology Group, Weill Cornell Medicine-Qatar, Cornell University, Qatar Foundation - Education City, P.O. Box 24144, Doha, Qatar; 3 Department of Healthcare Policy and Research, Weill Cornell Medicine, Cornell University, New York City, NY USA; 4 College of Health and Life Sciences, Hamad bin Khalifa University, Doha, Qatar

**Keywords:** United States, Herpes simplex virus type 1, Oral herpes, Genital herpes, Prevalence, Incidence, Mathematical model

## Abstract

**Background:**

Herpes simplex virus type 1 (HSV-1) is a prevalent lifelong infection that appears to be undergoing an epidemiologic transition in the United States (US). Using an analytical approach, this study aimed to characterize HSV-1 transitioning epidemiology and estimate its epidemiologic indicators, past, present, and future.

**Methods:**

An age-structured mathematical model was developed to describe HSV-1 transmission through oral and sexual modes of transmission. The model was fitted to the National Health and Nutrition Examination Surveys, 1976–2016 data series.

**Results:**

HSV-1 seroprevalence was projected to decline from 61.5% in 1970 to 54.8% in 2018, 48.5% in 2050, and 42.0% in 2100. In < 3 decades, seroprevalence declined by > 30% for those aged 0–19 years, but < 5% for those aged > 60. Meanwhile, the number of new infections per year (oral and genital) was persistent at 2,762,000 in 1970, 2,941,000 in 2018, 2,933,000 in 2050, and 2,960,000 in 2100. Of this total, genital acquisitions contributed 252,000 infections in 1970, 410,000 in 2018, 478,000 in 2050, and 440,000 in 2100—a quarter of which are symptomatic with clinical manifestations. For those aged 15–49 years, nearly 25% of incident infections are genital. Most genital acquisitions (> 85%) were due to oral-to-genital transmission through oral sex, as opposed to genital-to-genital transmission through sexual intercourse.

**Conclusion:**

HSV-1 epidemiology is undergoing a remarkable transition in the US, with less exposure in childhood and more in adulthood, and less oral but more genital acquisition. HSV-1 will persist as a widely prevalent infection, with ever-increasing genital disease burden.

**Electronic supplementary material:**

The online version of this article (10.1186/s12916-019-1285-x) contains supplementary material, which is available to authorized users.

## Introduction

Herpes simplex virus type 1 (HSV-1) is a highly contagious and lifelong infection, with high prevalence and rapid acquisition during childhood [1]. It is estimated that there were 118 million new infections globally in 2012 [1], much higher than that of HSV-2 infection at 19 million [2]. HSV-1 infection is never cleared and is endemic globally [3–5]. The virus is typically transmitted through contact with cold sores or via contact with oral secretions during asymptomatic shedding, such as when children share utensils or food [6]. Symptomatic infection is often characterized by oral or facial lesions at the initial portal of entry [7]. The infection can cause a spectrum of diseases, such as herpetic whitlow, gingivostomatitis, meningitis, encephalitis, and corneal blindness [8, 9].

HSV-1 can also be transmitted (during asymptomatic or symptomatic shedding) through oral sex or sexual intercourse, resulting in genital herpes, given the genital portal of entry [6, 7, 10, 11]. While HSV-2 infection is more transmissible sexually from males to females than from females to males [2, 12], current evidence cannot distinguish such sex differential for HSV-1 infection [13, 14]. There is evidence indicating that the proportion of first-episode genital herpes due to HSV-1 has increased steadily in industrialized countries [14–18], resulting in HSV-1 becoming recognized as a key sexually transmitted infection (STI) [19]. While HSV-2 antibody prevalence (seroprevalence) continues to decline in the United States (US) [7], and thus genital herpes due to HSV-2 is declining, emerging data suggest that genital herpes due to HSV-1 is increasing [20, 21]. In a study of college students in the US, the percentage of genital herpes attributed to HSV-1 (as opposed to HSV-2) increased from 31% in 1993 to 78% in 2001 [20]. A recent cohort study reported that nearly 60% of new genital herpes cases were attributed to HSV-1 [21, 22]. Similar trends have been observed in other industrialized countries [15–18].

With the improvements in hygiene and living conditions, HSV-1 seroprevalence appears to be declining in Western countries [1, 7, 23–29]. Seroprevalence in the US decreased from 42.6% in 1976–1980 to 30.1% in 2005–2010 in youth aged 14–19 years old [30]. A growing proportion of adolescents are reaching sexual debut lacking protective antibodies against acquisition of HSV-1 through oral sex or sexual intercourse [23, 30]. The larger proportion of unexposed youth, combined with an apparent increase in oral sex [31, 32], appears to have led to higher incidence of HSV-1-caused genital herpes [4].

Against this background, we aimed to quantitatively and analytically characterize the level and trend of the HSV-1 epidemiological transition, from an oral to increasingly genital infection in the US. We further aimed to forecast HSV-1 transmission dynamics over the coming decades. Specifically, we assessed the temporal evolution and varying age distribution of HSV-1 seroprevalence, HSV-1 oral and genital herpes prevalence, annual number of new infections, incidence rate of orally acquired versus genitally acquired HSV-1, and the contribution of oral versus genital acquisition to HSV-1 epidemiology. Thus, we aspired to provide a comprehensive characterization of the HSV-1 epidemiological transition, past, present, and future.

Our overarching goal was to inform public health response and ongoing efforts of HSV preventive and therapeutic vaccine development that are more focused on HSV-2, but increasingly interested on an HSV-1 vaccine as well [33, 34], by describing the patterns of oral and genital HSV-1 infections.

## Materials and methods

### Mathematical model

A deterministic dynamical model was constructed to describe the oral and sexual HSV-1 transmission in a given population—in this case, the US population (Additional file 1: Figure S1). The model was structured based on current understanding of the natural history and epidemiology of this infection. The model consisted of a set of coupled nonlinear differential equations that stratify the population into compartments according to age, HSV-1 status and stage of infection, and level of oral or sexual risk of exposure. The model was coded and analyzed in MATLAB R2016b [35].

The model accommodated two forms of exposure to HSV-1 by initial portal of entry. Individuals can be exposed orally leading to symptomatic or asymptomatic infection—both labeled thereon as *oral herpes* infection. They can also be exposed genitally leading to symptomatic or asymptomatic infection—both labeled thereon as *genital herpes* infection. Oral herpes can occur as a result of oral-to-oral transmission (believed to be the main mode of transmission) or genital-to-oral transmission (through oral sex). Genital herpes can occur as a result of oral-to-genital transmission (through oral sex) or genital-to-genital transmission (through sexual intercourse).

HSV-1 natural history in the model varied by form of exposure (oral versus genital herpes) and included the two main stages of HSV-1 shedding (through primary infection or reactivations) and no shedding (while in latency). Primary infection and reactivations were assumed equally transmissible, with an average infectiousness. Susceptible individuals (regardless of HSV-2 status) who acquired HSV-1 for the first time developed primary infection followed by latent infection and reactivations throughout a lifetime. Of note is that primary infection is often more symptomatic and severe than reactivations [10–12]. Those in latent infection episodically reactivated their infection, whether oral or genital, and shed the virus during this reactivation (Additional file 1: Figure S1). HIV status was not incorporated into the model, and thus did not affect HSV-1 natural history.

The model disaggregated the US population into 20 age groups, with each group representing a 5-year age band (0–4, 5–9, …, 95–99 years old). We assumed variation in risk of exposure to HSV-1 infection by age. While we did not incorporate behavioral heterogeneity in the oral (non-sexual) risk of exposure (other than by age), we assumed behavioral heterogeneity in the sexual risk of exposure for each age group. Each age group was divided into three sexual risk groups representing lower to higher risk based on the number of sexual partners over the past 12 months. The distribution of sexual risk behavior followed a power-law function, as informed by earlier data and modeling work [36–38], with the age dependence determined by sexual partnership data of the National Health and Nutrition Examination Survey (NHANES) [39].

The mixing between populations in the different age groups and risk groups, for the different modes of transmission, was dictated by mixing matrices that allow a range of mixing behaviors varying from fully assortative mixing (mixing only with individuals in the same age or risk group) to fully proportionate mixing (mixing with individuals with no preferential bias for a specific age or risk group) [40–42].

Details of model structure are in Additional file 1: Supplemental Information 1.

### Model parameterization

Model parameters were chosen based on current empirical data on HSV-1 natural history and epidemiology and are listed in Additional file 1: Table S2 along with their justifications and references. The model was applied to ten biennial rounds of the nationally representative and population-based NHANES survey (for the non-institutionalized US population) that included HSV-1 seroprevalence data from 1976 to 2016 [39]. All surveys followed a standardized methodology, both analytically and in laboratory procedures [39]. Sampling, for each survey, was first conducted by randomly selecting neighborhoods (strata) from geographic divisions/counties (primary sampling units), followed by a random selection of households from these neighborhoods [39]. Eligible inhabitants of those households were then interviewed, and a subsample was tested for glycoprotein specific to HSV-1 (designated gG-1) and to HSV-2 (designated gG-2) in sera using solid-phase enzymatic type-specific immunodot assays [39]. Demographic, sexual, and HSV laboratory examination data files for each round were extracted, merged, and analyzed following NHANES standardized “survey methods and analytic guidelines” [43]. Sampling weights were applied to all NHANES-derived measures.

**Fig. 1:**
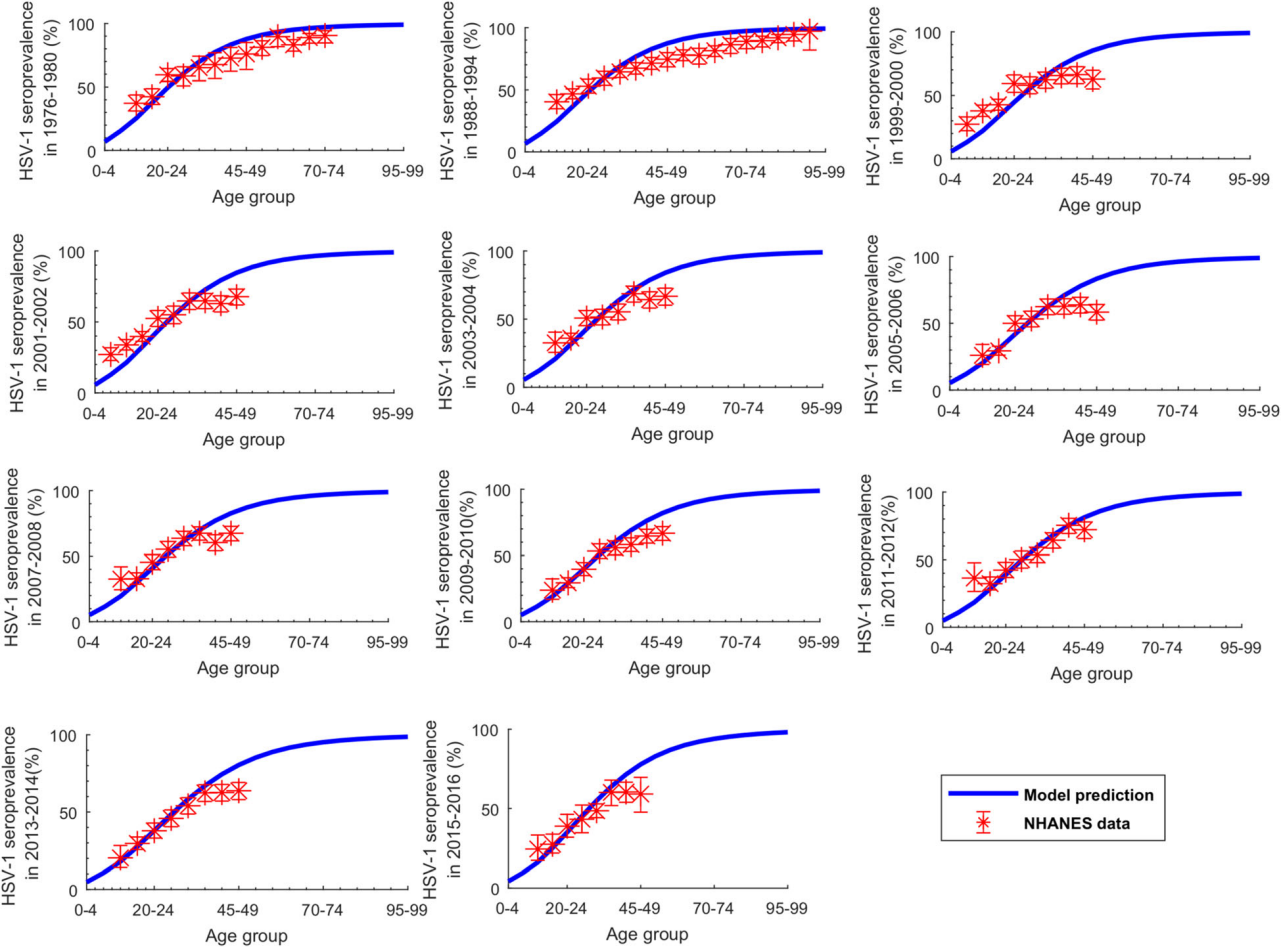
Fitting of the age-specific distribution of HSV-1 seroprevalence in the US. The fitted HSV-1 seroprevalence in each 5-year age band, compared to the National Health and Nutrition Examination Surveys (NHANES) data from 1976 up to 2016

**Fig. 2:**
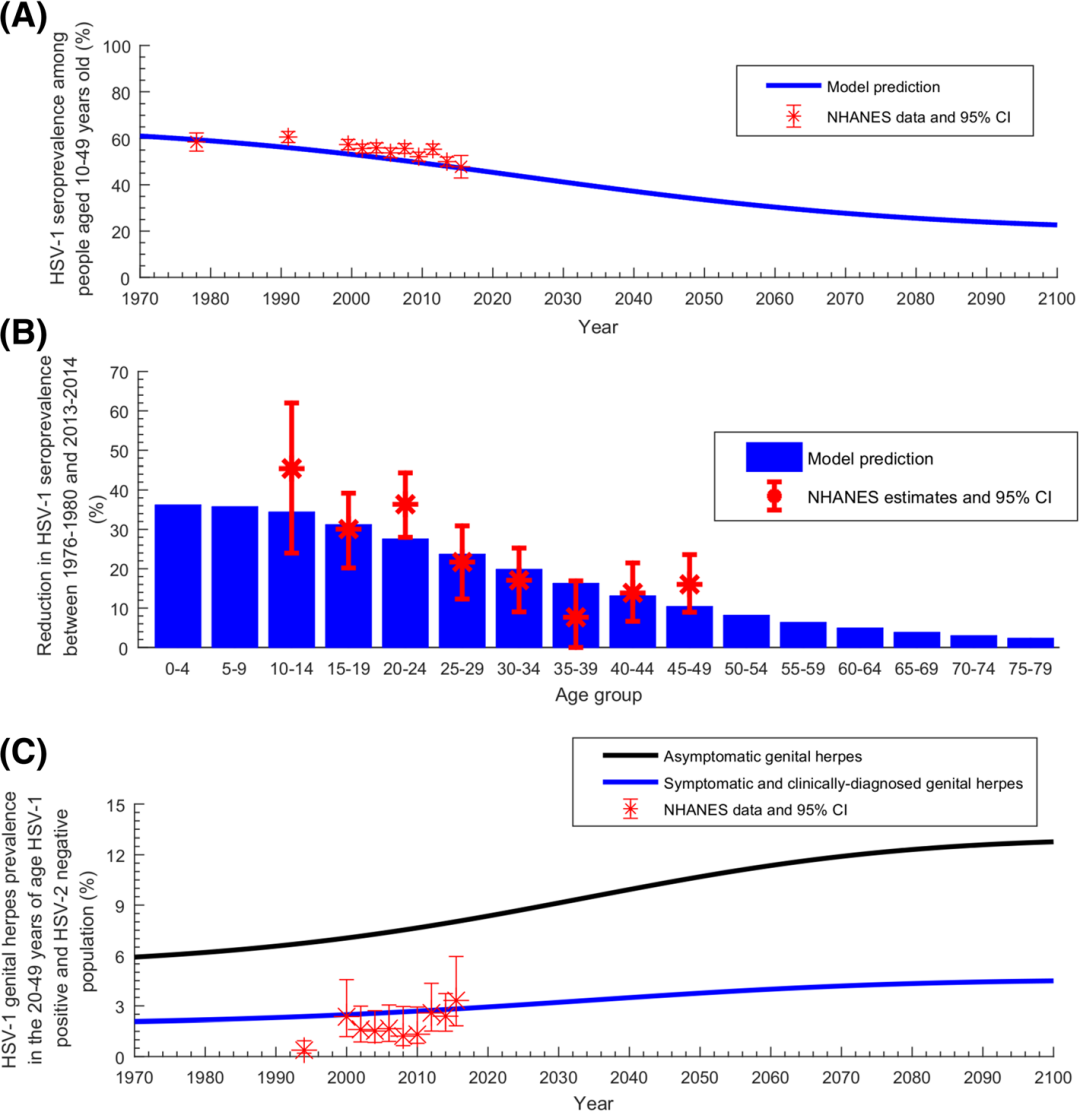
Temporal evolution of HSV-1 seroprevalence in the US. **a** Estimated HSV-1 seroprevalence in people aged 10–49 years, compared to the National Health and Nutrition Examination Surveys (NHANES) data. **b** Estimated reduction in HSV-1 seroprevalence per age group between 1976–1980 and 2013–2014, compared to the measured reduction in NHANES data. **c** Estimated asymptomatic as well as ever-symptomatic and clinically diagnosed genital herpes prevalence in *HSV-1-antibody-positive* and *HSV-2-antibody-negative* 20–49 years old population—the latter compared to self-reported NHANES data. HSV-1 *genital herpes* is defined as any HSV-1 infection acquired genitally, regardless of presence or absence of disease or clinical manifestations

For each NHANES round, we derived the age-specific distribution of HSV-1 seroprevalence (Fig. 1 and Fig. 2a), and the age-specific distribution of self-reported *ever-symptomatic and clinically diagnosed genital herpes* prevalence in *concurrently* HSV-1-antibody-positive and HSV-2-antibody-negative individuals (Fig. 2c and Additional file 1: Figure S4)—to distinguish HSV-1 from HSV-2 genital herpes. The latter measure was derived using the NHANES question of “has a doctor or other health care professional ever told you that you had genital herpes?”

For the sexual-behavior parametrization, we derived the distribution (for all ages) of the reported number of sexual partnerships in the past 12 months from the 1999–2014 NHANES rounds. For each round, the distribution of the reported number of heterosexual partners (0 partner, 1 partner, or 2 or more partners) was derived by combining, respectively, men and women answers to the question “in the past 12 months, with how many women/men have you had sex?”. Estimates for the population proportion of individuals having no sexual partner, one partner, and two or more partners over the past 12 months were then generated by pooling the latter measures across NHANES rounds using a DerSimonian-Laird random-effects model [44] with inverse variance weighting.

The *age-specific* distribution of sexual partnerships in the past 12 months (that is how many partners were reported in each age group) was extracted from the 2013–2014 NHANES round. The overall level of oral-sex risk behavior in the US population was determined through model fitting to data. The overall level of sexual-intercourse risk behavior was derived by adapting an earlier model for HSV-2 transmission [12] and fitting it to NHANES data for HSV-2 infection [39].

US demographics and trends (Additional file 1: Figure S3) were obtained from the United Nations’ World Population Prospects database [45].

### Model fitting

The model was fitted to NHANES time and age series data for HSV-1 seroprevalence. The model was also fitted to NHANES data for the self-reported ever-symptomatic genital herpes prevalence among HSV-1-antibody-positive and HSV-2-antibody-negative individuals—that is among individuals who have not acquired HSV-2 infection. The latter fitting was incorporated assuming that risk of exposure to HSV-1 is independent from risk of exposure to HSV-2. All data fitting was performed using a nonlinear least-square fitting method, by minimizing the sum of squares between all data points and model predictions, using the Nelder-Mead simplex algorithm [46]. To fit the model to trend data, the overall population-level oral-to-oral risk of exposure was allowed to vary.

Further details on data sources and model fitting is in Additional file 1: Supplemental Information 2.

### Uncertainty and sensitivity analyses

A multivariable uncertainty analysis was conducted to specify the range of uncertainty around the epidemic projections, by deriving the 95% uncertainty intervals (UIs) [42, 47] around model outcomes. The 95% UIs were derived by implementing 500 runs of the model applying Latin Hypercube sampling from a multidimensional distribution of the model parameters (Additional file 1: Table S1)—assuming ± 30% uncertainty around the parameters’ point estimates, as informed by existing mathematical modeling approaches [42, 48–50]. In each run, the parameters’ values were randomly selected from their specified ranges, and the model was refitted to data. The means for each predicted model outcome over these runs were calculated at each time point. The 95% UIs for each predicted model outcome at each time point were determined using the 2.5th and 97.5th percentiles of the 500 runs.

Given the range of available data for oral HSV-1 shedding frequency [6, 10, 11], we conducted a sensitivity analysis to assess the impact of extreme variations in the oral HSV-1 shedding frequency, which ranged between 5 and 18% [6, 10, 11]. Similarly, we conducted a sensitivity analysis to assess the impact of extreme variations in the genital HSV-1 shedding frequency, which ranged between 0.5 and 6% [11, 51].

## Results

Figure 1 shows the model fits to the age-specific HSV-1 seroprevalence for the different years of the NHANES rounds. Figure 2a shows the model fit to HSV-1 seroprevalence, and Fig. 2c shows the model fit to the ever-symptomatic genital herpes prevalence—all for the same NHANES rounds. Additional file 1: Figure S3 shows the model fit to the US population size. The model produced robust fits to all of these data, but slightly tended to overestimate seroprevalence in those > 40 years old (Fig. 1).

The model-predicted trends for HSV-1 seroprevalence are seen in Fig. 2. In Fig. 2a, the model-predicted trend for seroprevalence in the *10–49 years old population* indicates a trajectory of declining seroprevalence from 61.1% in 1970, down to 46.2% in 2018, 33.5% in 2050, and 22.7% in 2100. In the *total population*, seroprevalence declined from 61.5% in 1970, to 54.8% in 2018, 48.4% in 2050, and 42.1% in 2100 (not shown in figure). Figure 2b provides the model-predicted reduction in seroprevalence between 1976 and 1980 and 2013–2014—in agreement with the NHANES-measured reduction. The figure predicts > 30% reduction in seroprevalence over this timeframe for those aged 0–19 years, but < 5% reduction for those > 60.

In Fig. 2c, the model-predicted ever-symptomatic genital herpes prevalence in *HSV-1-positive HSV-2-negative* 20–49 years old population was projected to increase steadily (but slowly) from 2.9% in 2018 up to 3.8% in 2050 and 4.5% in 2100. The asymptomatic genital herpes prevalence was further projected to increase steadily from 8.2% in 2018 up to 10.7% in 2050 and 12.8% in 2100.

**Fig. 3:**
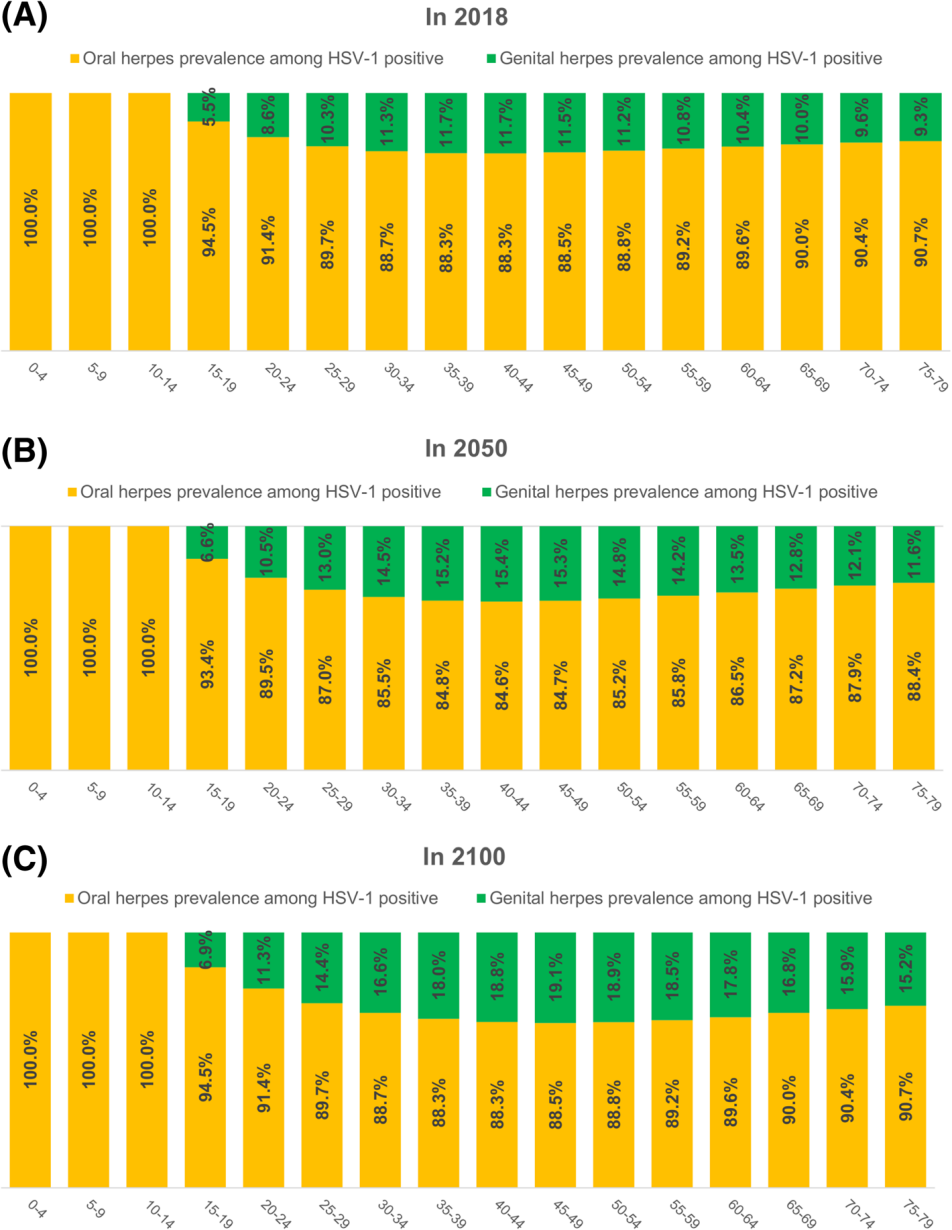
Age-specific relative contribution of orally acquired versus genitally acquired HSV-1 among *prevalent* HSV-1 infections in the US. Estimated age-specific distribution of oral herpes prevalence versus genital herpes prevalence in the *total HSV-1-antibody-positive population* in 2018 (**a**), 2050 (**b**), and 2100 (**c**). HSV-1 *oral herpes* is defined as any HSV-1 infection acquired orally, regardless of the presence or absence of disease or clinical manifestations. HSV-1 *genital herpes* is defined as any HSV-1 infection acquired genitally, regardless of the presence or absence of disease or clinical manifestations

Figure 3 shows the age-specific relative contribution of originally orally acquired versus genitally acquired HSV-1 in the *HSV-1-positive population* (that is among *prevalent/existing* infections) in 2018 (Fig. 3a), 2050 (Fig. 3b), and 2100 (Fig. 3c). The contribution of genital acquisition increased with age following sexual debut up to 30–49 years of age, but then declined at older ages. Across sexually active age groups, the contribution ranged between 5.5 and 11.7% in 2018, 6.6 and 15.4% in 2050, and 6.9 and 19.1% in 2100. The contribution in the total HSV-1-positive population of all ages was projected to increase steadily from 9.9% in 2018 up to 12.6% in 2050 and 15.7% in 2100 (not shown in this figure).

**Fig. 4:**
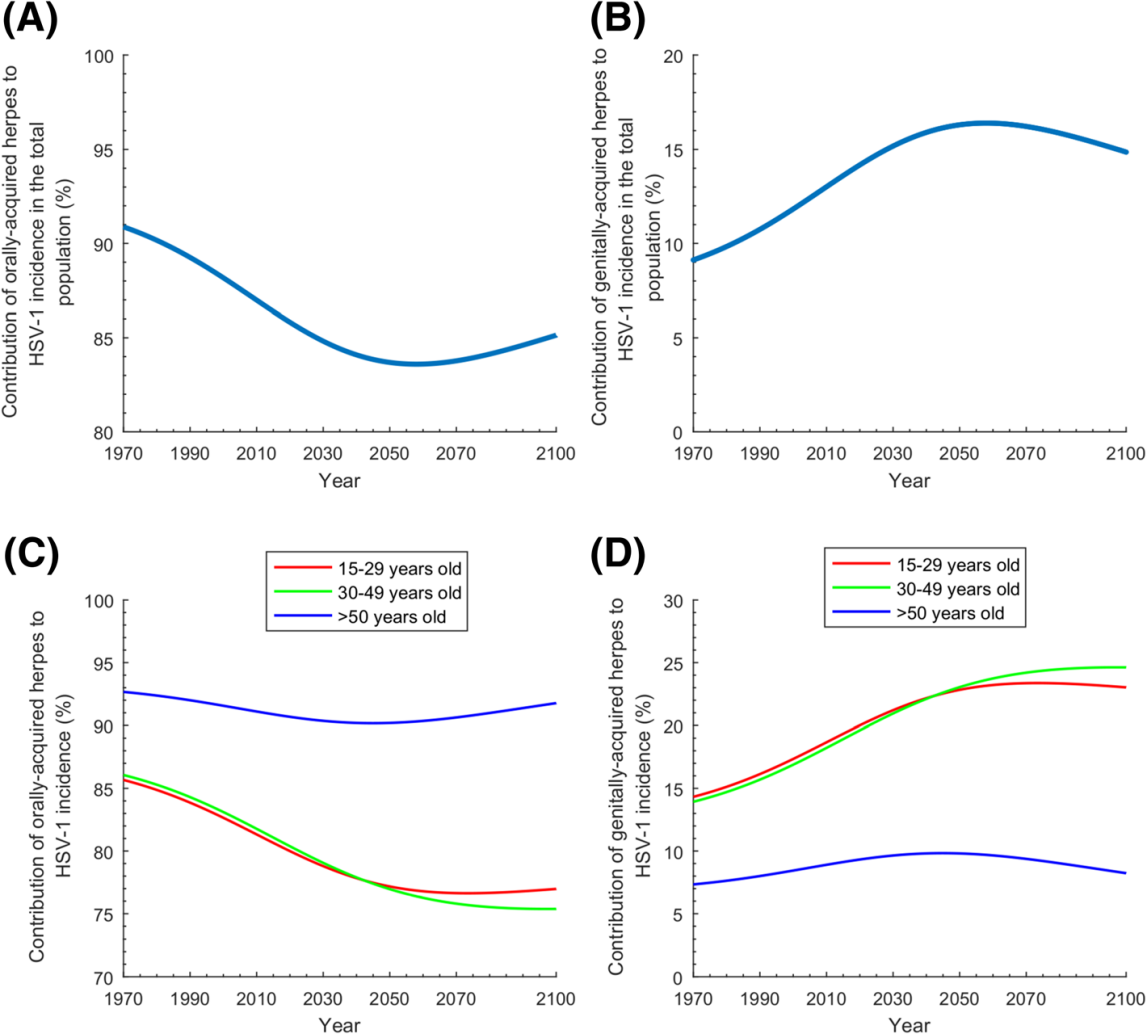
Relative contribution of oral herpes versus genital herpes to HSV-1 incidence in the US. **a**, **b** Contribution of orally acquired versus genitally acquired HSV-1 among new (incident) infections in the total population of all ages. **c**, **d** Contribution of orally acquired versus genitally acquired HSV-1 among new infections in those aged 15–29, 30–49, and > 60 years. Of note is that the different panels have different *y*-axis scales

The model-predicted trends for the relative contribution of oral and genital herpes to HSV-1 incidence are seen in Fig. 4. Figure 4a and Fig. 4b show the time trend of the relative contribution of orally acquired versus genitally acquired HSV-1 among new (*incident*) infections in *the total population*. Figure 4c and Fig. 4d show these time trends but for *three relevant age brackets*. In the total population, genital acquisition in incident infections increased (and oral acquisition decreased) steadily starting from 1970 up to 2060 and peaked at 16.4% (Fig. 4a and Fig. 4b), after which the trend (slowly) reversed. Additional file 1: Figure S2 and its discussion in Additional file 1: Supplemental Information 3 explain the steady increase in genital acquisition, leading eventually to a decrease, which was found to be a consequence of a *turning point* in the infection dynamics, which occurs as HSV-1 seroprevalence declines down to below 50%.

The contribution of oral versus genital acquisition to incidence varied by age bracket (Fig. 4c and Fig. 4d). For the 15–29 and 30–49 years old brackets, genital acquisition increased until about 2060 and then stabilized at nearly 25% of incident infections. Meanwhile, for those > 50, genital acquisition increased up to 2045 peaking at 9.8%, and then slowly declined.

**Fig. 5:**
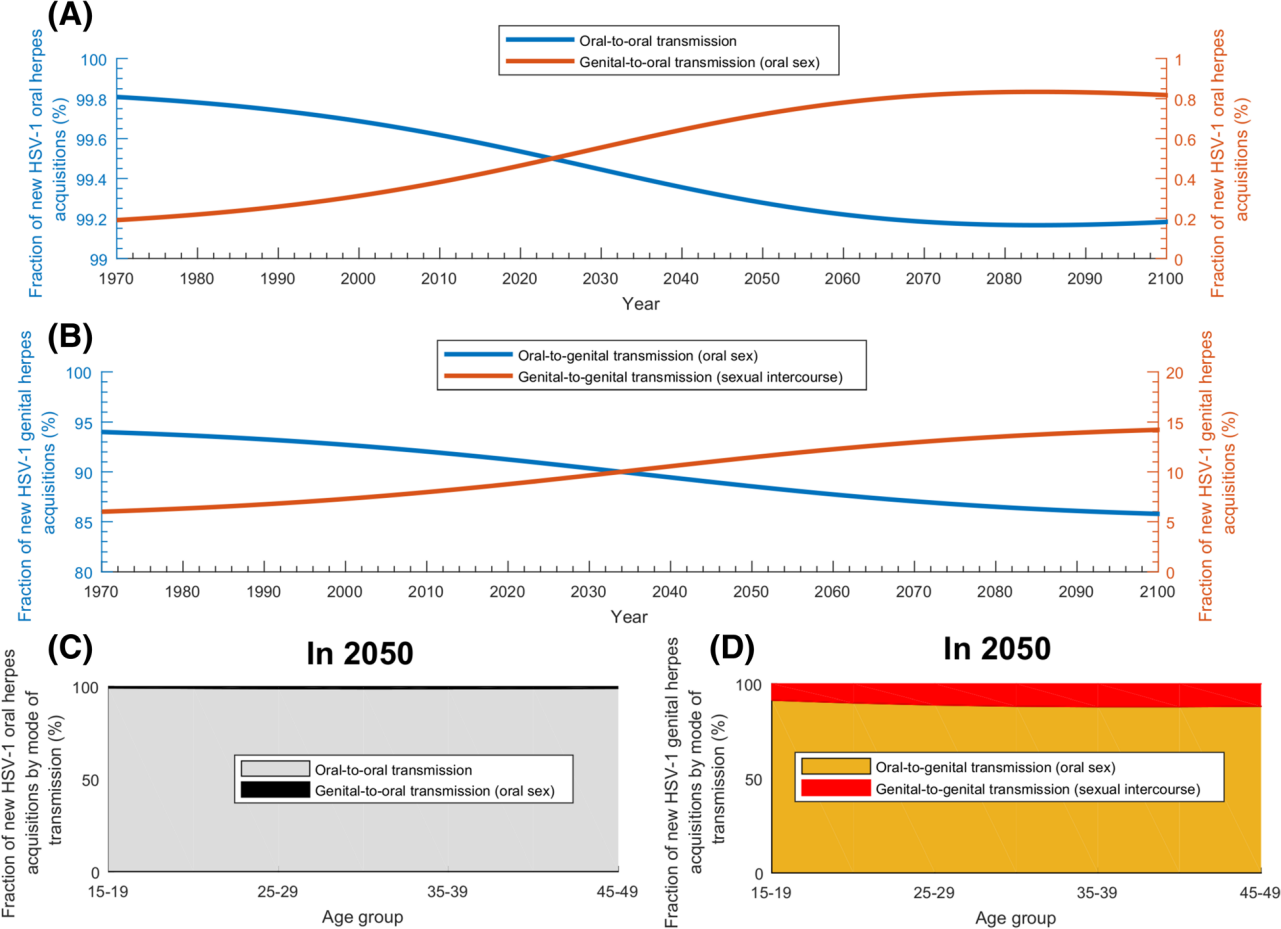
Relative contribution of the different HSV-1 modes of transmission to HSV-1 incidence in the US. **a** Projected contribution to HSV-1 oral herpes incidence of *oral-to-oral* transmission versus *genital-to-oral* (through oral sex) transmission. **b** Projected contribution to HSV-1 genital herpes incidence of *oral-to-genital* (through oral sex) transmission versus *genital-to-genital* (through sexual intercourse) transmission. **c** New HSV-1 oral herpes acquisitions by mode of transmission versus age in 2050. **d** New HSV-1 genital herpes acquisitions by mode of transmission versus age in 2050. HSV-1 *oral herpes* is defined as any HSV-1 infection acquired orally, regardless of the presence or absence of disease or clinical manifestations. HSV-1 *genital herpes* is defined as any HSV-1 infection acquired genitally, regardless of the presence or absence of disease or clinical manifestations

Figure 5 shows the relative contribution of the different HSV-1 modes of transmission to incidence. Nearly all (> 99%) of oral acquisitions for all times (Fig. 5a) and ages (Fig. 5c) were due to oral-to-oral transmission. Genital-to-oral transmission (through oral sex), despite being increasing with time, was always negligible and peaked at only 0.8% around 2090.

As for genital acquisition, oral-to-genital transmission (through oral sex) was dominant for all times (Fig. 5b) and ages (Fig. 5d), but with an appreciable genital-to-genital transmission (through sexual intercourse). Oral-to-genital transmission declined with time from 94.3% in 1970, to 91.7% in 2018, 88.9% in 2050, and 85.9% in 2100. Meanwhile, genital-to-genital transmission increased from 5.7% in 1970 to 8.3% in 2018, 11.1% in 2050, and 14.1% in 2100. By age in 2050, genital-to-genital transmission was mostly around 10–12% for most sexually active age groups (Fig. 5d).

**Fig. 6:**
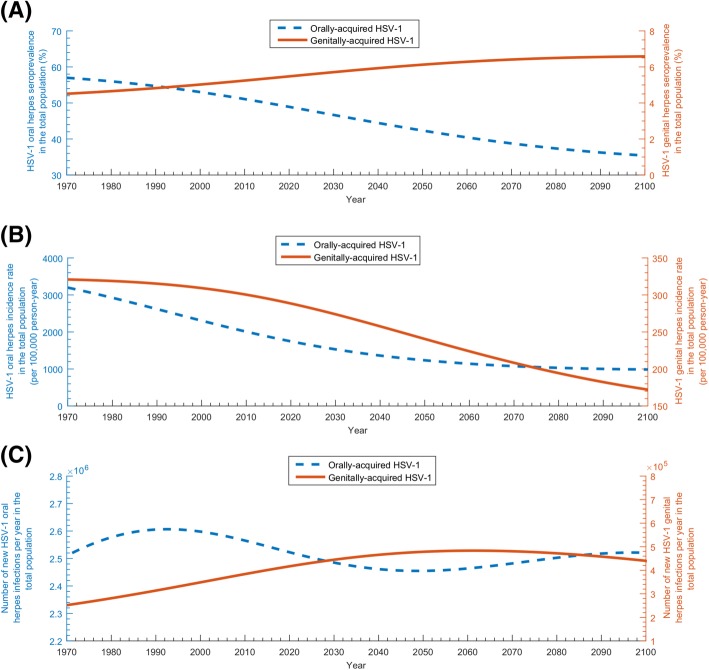
Temporal evolution of key epidemiologic indicators of HSV-1 infection in the total population of all ages of the US. **a** Estimated orally acquired HSV-1 seroprevalence versus genitally acquired HSV-1 seroprevalence. **b** Estimated orally acquired HSV-1 incidence rate versus genitally acquired HSV-1 incidence rate. **c** Estimated annual number of new (incident) orally acquired versus genitally acquired HSV-1 infections. Of note that the *y*-axis scales are different for the oral versus genital estimates

The model-predicted trends for key epidemiological indicators of HSV-1 infection are seen in Fig. 6. Figure 6a shows the orally acquired seroprevalence versus the genitally acquired seroprevalence in the total US population (noting that HSV-1 seroprevalence in the total population is the sum of the orally acquired and genitally acquired seroprevalence). Orally acquired seroprevalence decreased starting from 1970 onwards—it was 57.0% in 1970, 49.4% in 2018, 42.3% in 2050, and 35.5% in 2100. Genitally acquired seroprevalence increased from 1970 onwards—it was 4.5% in 1970, 5.4% in 2018, 6.1% in 2050, and 6.6% in 2100. Additional file 1: Figure S4A shows similar trends for only adults 15–59 years of age.

Figure 6b shows the declining trends for *HSV-1 incidence rate*, for each of orally acquired and genitally acquired herpes. Incidence rate for orally acquired herpes (per 100,000 person-year) was 3197 in 1970, 1795 in 2018, 1235 in 2050, and 986 in 2100. Meanwhile, incidence rate for genitally acquired herpes (per 100,000 person-year) was 321 in 1970, 291 in 2018, 241 in 2050, and 172 in 2100. Total incidence rate (regardless of mode of acquisition and per 100,000 person-year) was 3518 in 1970, 2086 in 2018, 1476 in 2050, and 1158 in 2100. Additional file 1: Figure S4B shows overall similar trends for only adults 15–59 years of age.

Figure 6c shows the *annual number* of new (incident) orally acquired and genitally acquired herpes infections. Orally acquired incidence was 2,510,000 in 1970, 2,531,000 in 2018, 2,455,000 in 2050, and 2,520,000 in 2100. Meanwhile, genitally acquired incidence was 252,000 in 1970, 410,000 in 2018, 478,000 in 2050, and 440,000 in 2100. Total incidence was 2,762,000 in 1970, 2,941,000 in 2018, 2,933,000 in 2050, and 2,960,000 in 2100. Additional file 1: Figure S4C shows overall similar trends for only adults 15–59 years of age.

Additional file 1: Figure S5 shows the uncertainty analysis results for select relevant outcomes and projections. The uncertainty in the input parameters resulted in a range for the predicted outcomes. For example, the contribution of orally acquired HSV-1 among new infections varied in 2018 between 84.9 and 90.0% and in 2050 between 83.3 and 87.7%. Overall, the results of the uncertainty analysis affirmed model predictions and findings.

Additional file 1: Figure S6 and S7 show the results of the sensitivity analyses for select relevant outcomes and projections. Despite wide variations in the oral and genital HSV-1 shedding frequencies, the sensitivity analyses supported overall our conclusions, with most variations in projected outcomes being evident after 2050.

## Discussion

Using an analytical modeling approach, we characterized a progressively transitioning epidemiology of HSV-1 infection from its historical prototype, where the infection was propagating by oral-to-oral transmission mostly during childhood, into a complex epidemiologic dynamics sustained by different modes of transmission, affecting different age cohorts, in different ways and times. Our results demonstrate a more subtle and intriguing transition than previously thought, with oral sex playing an important role in infection transmission—HSV-1 is earning its status as a key STI.

Seroprevalence and incidence rate were predicted to decline for decades to come, with ever-decreasing exposure during childhood (Fig. 2a and Fig. 6b). In less than three decades, seroprevalence declined by > 30% among those < 15 years of age (Fig. 2b)—consistent with living conditions in childhood playing an important role in infection risk [30]. Nonetheless, absolute incidence (number of new infections per year) will not decline appreciably—demographic growth with ever-increasing number of susceptibles will sustain high incidence at about 3 million new infections every year (Fig. 6c). HSV-1 will remain a widely prevalent infection in the US population.

HSV-1 oral herpes seroprevalence was projected to decline by 14% between 2018 and 2050 (Fig. 6a). Although oral acquisition (versus genital acquisition) will decline for decades to come (Fig. 4a), oral herpes will remain the dominant form of infection, with about 2.5 million new oral acquisitions every year (Fig. 6c). The decline in oral herpes will be more pronounced for children, as more children will age and reach sexual debut with no protective antibodies against HSV-1 genital acquisition.

Meanwhile, HSV-1 genital herpes will continue to grow for decades (Fig. 4b), with age being a determining factor in exposure risk (Fig. 4d). Close to 500,000 new genital acquisitions will occur every year for decades (Fig. 6c). A quarter of these will be symptomatic, which in context of emerging evidence [3, 21, 52–54], will result in significant clinical and psychosocial morbidity. Young adults will carry the largest burden—25% of acquisitions will be genital in the 15–49 age group (Fig. 4d). The increasing number of genital HSV-1 infection in women of childbearing age could lead to an increase in the incidence of neonatal herpes, a serious disease outcome with high risk of mortality [55]. HSV-1 genital herpes seroprevalence will reach 7% in the US population (Fig. 6a), constituting about 15% of all prevalent infections (Fig. 3). The growth in incidence will not be sustainable, however, reaching its peak around 2060 and then slowly declining (Fig. 4b and Fig. 6c).

The intriguing growth but then saturation of genital herpes was found to reflect the subtle dynamics of the oral-to-genital mode of acquisition. This mode of acquisition, occurring through oral sex between an orally infected seropositive person and a susceptible seronegative person, is driven not only by the increasingly larger population reaching sexual debut uninfected, but critically by the *large reservoir* of orally acquired infections. Additional file 1: Supplemental Information 3 provides a heuristic explanation of this subtle effect leading to a key *turning point* in HSV-1 epidemiology—genital herpes will peak as seroprevalence declines and approaches the 50% landmark. This is in essence what has been unfolding in the US in recent decades: HSV-1 genital herpes has been rapidly increasing because seroprevalence has been approaching the 50% turning point. The declining seroprevalence has been leading to a larger susceptible young population, and growing genital herpes incidence, thanks to the large pool of orally acquired prevalent infections. Eventually, this same declining seroprevalence will yield (to the contrary) to declining genital herpes incidence, as the pool of orally acquired prevalent infections shrinks to an extent that it can no longer sustain as much oral-to-genital transmission through oral sex.

This finding presents a cautionary tale. Since seroprevalence is very high in most global regions [1, 13, 14], but potentially declining with the improvements in living conditions, we should brace ourselves for a large increase in HSV-1 genital herpes, *if* the declining seroprevalence will approach the 50% turning point. Growing evidence from different, mostly Western countries, suggests increasing HSV-1 genital herpes trends [1, 15–18, 23, 56, 57]. However, it remains to be investigated whether other countries or global regions are experiencing such a transition in HSV-1 epidemiology, as characterized here for the US.

The four modes of transmission contributed to some extent or another to HSV-1 incidence (Fig. 5). However, oral-to-oral transmission will remain the dominant mode, but with increasing role for the sexual modes of transmission. Though both oral-to-genital (oral sex) and genital-to-genital (sexual intercourse) modes of transmission will steadily increase, the genital-to-genital mode will increase faster, as the *pool* of HSV-1 genital herpes infections increases. Currently, < 10% of HSV-1 genital herpes is due to genital-to-genital transmission, but this will increase up to 15% shortly after 2050 (Fig. 5b). Notably, genital-to-oral transmission will remain negligible explaining < 1% of oral acquisitions (Fig. 5a).

Limitations may have affected this study. The model produced robust fits for the empirical data, despite a slight overestimation for the elderly age groups (Fig. 1). Model projections can depend on model structure and quality of input data. Future projections were generated by fitting the model to past and current data, but future incidence can be influenced by factors that are difficult to predict at present. To reduce complexity, sex was not included explicitly in the model, nor did the model distinguish between vaginal intercourse versus anal intercourse. We assumed that HSV-1 infectiousness does not vary by presence of clinical symptoms, but this is unlikely to appreciably affect our results, as most shedding is asymptomatic anyway [4, 6, 11, 58]. We assumed that individuals who acquire HSV-1 orally are protected against (or have a negligible risk of) HSV-1 genital acquisition. While this assumption appears to be supported by current evidence [14, 51], it remains to be seen whether there is an appreciable acquisition of non-primary HSV-1 genital herpes.

We assumed that HSV-1 transmission probability per act of sexual intercourse is equal to that of HSV-2, but the validity of this assumption is uncertain. If HSV-1 is found less infectious *per act* than HSV-2, this will reduce the genital-to-genital mode of transmission. Moreover, we assumed that oral and genital HSV-1 shedding continues at a fixed frequency, but recent data, particularly for HSV-1 genital shedding frequency, suggests that shedding declines with time post-infection [51]. Accordingly, it is possible that we could be overestimating the already relatively small genital-to-genital mode of transmission.

The oral HSV-1 shedding frequency was based on most current data [6], but earlier studies suggested a lower shedding frequency [10, 11]. If oral HSV-1 shedding frequency is indeed lower, the conducted sensitivity analysis (Additional file 1: Figure S6) suggests that both oral and genital HSV-1 incidence would be lower, particularly so for the long-term predictions—otherwise our findings are largely invariable. The genital HSV-1 shedding frequency was based on data collected from HSV-1 and HSV-2 seropositive individuals [11], but a recent study (only on HSV-1 seropositive individuals) suggested a higher genital HSV-1 shedding frequency, though the shedding did not persist for a long time post-infection [51]. If genital HSV-1 shedding frequency is persistently higher, the conducted sensitivity analysis (Additional file 1: Figure S7) suggests that we could have underestimated the genital-to-genital acquisition.

Our study has strengths. We used an elaborate mathematical model to capture the complex HSV-1 transmission dynamics, and the model was anchored on current and quality data for HSV-1 natural history and transmission parameters. This is (to our knowledge) the first such study to model the intricate interplay of HSV-1’s four modes of transmission, and at a level of detail that is well beyond what is amenable to empirical epidemiologic studies. A major strength of this analysis is that it is grounded on quality and standardized population-based data, that of NHANES [39], and provides estimates that are representative of the socio-demographic and mode of acquisition diversity in the population at large. The model was parametrized by NHANES data of HSV-1 seroprevalence for four decades. Remarkably, with such robust input data, the model parameters are well-constrained limiting the uncertainty around model results. Indeed, model outcomes fitted the empirical data, and the predicted trends matched actual trends.

## Conclusion

Epidemiology of HSV-1 infection in the US is undergoing a remarkable and subtle transition, with less exposure in childhood and more in adulthood, and less oral acquisition but more genital acquisition. Though seroprevalence will decline for several decades, incidence will persist at 3 million new infections every year. Of this total, close to 500,000 will be genital acquisitions, mainly through oral sex. HSV-1 will persist as a major cause of first-episode genital herpes for decades to come. Young adults will be most affected, with 25% of HSV-1 incidence among them being genital. The rise of HSV-1 genital herpes will peak shortly after 2050, as seroprevalence in the total population delves below 50%, a key turning point in HSV-1 epidemiology.

These findings indicate endurance of considerable HSV-1 disease burden in the US, with ever increasing genital disease burden. They also inform public health efforts about the scale of the disease burden and provide strategic data of estimates and projections. The results further inform decisions about counseling practices in clinical care, but more work is needed to develop specific counseling and clinical guidelines. Last but not the least, the findings demonstrate the need for continuous surveillance and argue for rapid development of a prophylactic vaccine to control transmission and to prevent associated clinical and psychosocial disease burden.

## Additional file

Additional file 1: Supplemental information including technical details about the mathematical model (Supplemental Information 1), the parameter values (Supplemental Information 2), and HSV-1 genital herpes 50% seroprevalence turning point (Supplemental Information 3). **Tables S1**-**S2.** **Figure S1**-**S7.** (DOCX 3158 kb)

## Abbreviations

HSV-1: Herpes simplex virus type 1
HSV-2: Herpes simplex virus type 2
NHANES: National Health and Nutrition Examination Survey
STI: Sexually transmitted infection
UI: Uncertainty interval
US: United States
