# Supplementary material for: Characterizing the transitioning epidemiology of herpes simplex virus type 1 in the USA: model-based predictions
Source: BMC Med. 2019 Mar 11;17:57. doi: 10.1186/s12916-019-1285-x (PMC6410528; doi:10.1186/s12916-019-1285-x)
Supplement: Supplementary file 1 — Supplemental information including technical details about the mathematical model (Supplemental Information 1), the parameter values (Supplemental Information 2), and HSV-1 genital herpes 50% seroprevalence turning point (Supplemental Information 3). Tables S1-S2. Figure S1-S7. (DOCX 3158 kb) [file 12916_2019_1285_MOESM1_ESM.docx]

**Supplemental Information**

Characterizing the Transitioning Epidemiology of Herpes Simplex Virus Type 1 in the United States: Model-Based Predictions

Houssein H. AYOUB, PhD^1,2,3^ Hiam CHEMAITELLY, MSc^2^ and Laith J. ABU-RADDAD, PhD^2,3,4^

^1^Department of Mathematics, Statistics, and Physics, Qatar University, Doha, Qatar

^2^Infectious Disease Epidemiology Group, Weill Cornell Medicine-Qatar, Cornell University, Qatar Foundation - Education City, Doha, Qatar

^3^Department of Healthcare Policy and Research, Weill Cornell Medicine, Cornell University, New York City, New York, USA

^4^College of Health and Life Sciences, Hamad bin Khalifa University, Doha, Qatar

Reprints or correspondence:

Dr. Houssein H. Ayoub, Department of Mathematics, Statistics, and Physics, Qatar University, P.O. Box 2713, Doha, Qatar. Telephone: +(974) 4403-7543. E-mail: [hayoub@qu.edu.qa](mailto:hayoub@qu.edu.qa).

Professor Laith J. Abu-Raddad, Infectious Disease Epidemiology Group, Weill Cornell Medicine in Qatar, Qatar Foundation - Education City, P.O. Box 24144, Doha, Qatar. Telephone: +(974) 4492-8321. Fax: +(974) 4492-8333. E-mail: [lja2002@qatar-med.cornell.edu](mailto:lja2002@qatar-med.cornell.edu).

**Supplemental Information 1: Mathematical model**

A deterministic mathematical model was developed to describe oral and genital herpes simplex virus type 1 (HSV-1) transmission in a population. An infection acquired orally was labelled as “oral herpes”, regardless of the presence or absence of disease or clinical manifestations. An infection acquired genitally was labelled as “genital herpes”, regardless of the presence or absence of disease or clinical manifestations. The model stratified the population into compartments according to age group, HSV-1 status and stage of infection, and level of risk of exposure.

**Fig S1.** Schematic diagram describing the structure of the HSV-1 model.


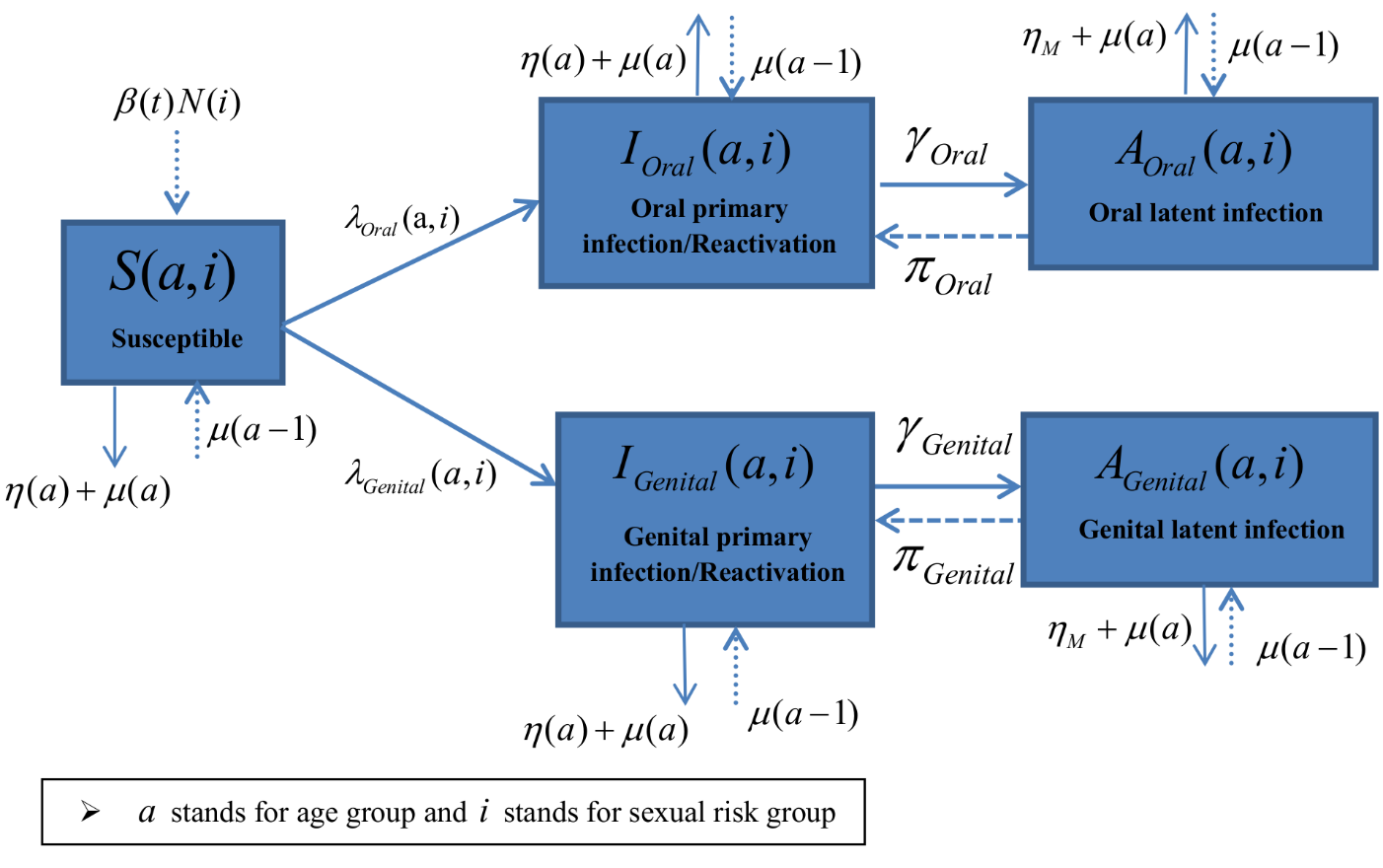


- 1. *Model equations and description*

The model was expressed in terms of a system of coupled nonlinear differential equations for each age group and sexual risk group:

Fully susceptible population:

$$\left\{\begin{matrix} \frac{dS(1,i)}{dt}=\beta(t)N(i)-\left(\eta(t,1)+\mu(1)+\lambda_{Oral}(1,i)\right)S(1,i) \\ 1<a\leq3: \\ \frac{dS(a,i)}{dt}=\mu(a-1)S(a-1,i)-\left(\eta(t,a)+\mu(t,a)+\lambda_{Oral}(a,i)\right)S(a,i) \\ a>3: \\ \frac{dS(a,i)}{dt}=\mu(a-1)S(a-1,i)-\left(\eta(t,a)+\mu(t,a)+\lambda_{Oral}(a,i)+\lambda_{Genital}(a,i)\right)S(a,i) \end{matrix}\right.$$

HSV-1 oral herpes infection:

$$\left\{\begin{matrix} \frac{dI_{Oral}(a=1,i)}{dt}=\lambda_{Oral}(1,i)S(1,i)-\left(\eta(t,1)+\mu(1)+\gamma_{Oral}\right)I_{Oral}(1,i)+\pi_{Oral}A_{Oral}(1,i) \\ \frac{dA_{Oral}(a=1,i)}{dt}=\gamma_{Oral}I_{Oral}(1,i)-\left(\eta(t,1)+\mu(1)+\pi_{Oral}\right)A_{Oral}(1,i) \\ a>1: \\ \frac{dI_{Oral}(a,i)}{dt}=\mu(a-1)I_{Oral}(a-1,i)+\lambda_{Oral}(a,i)S(a,i)-\left(\eta(t,a)+\mu(a)+\gamma_{Oral}\right)I_{Oral}(a,i)+\pi_{Oral}A_{Oral}(a,i) \\ \frac{dA_{Oral}(a,i)}{dt}=\mu(a-1)A_{Oral}(a-1,i)+\gamma_{Oral}I_{Oral}(a,i)-\left(\eta(t,a)+\mu(a)+\pi_{Oral}\right)A_{Oral}(a,i) \end{matrix}\right.$$

HSV-1 genital herpes infection:

$$\left\{\begin{matrix} a>3: \\ \frac{dI_{Genital}(a,i)}{dt}=\mu(a-1)I_{Genital}(a-1,i)+\lambda_{Genital}(a,i)S(a,i)-\left(\eta(t,a)+\mu(a)+\gamma_{Genital}\right)I_{Genital}(a,i)+\pi_{Genital}A_{Genital}(a,i) \\ \frac{dA_{Genital}(a,i)}{dt}=\mu(a-1)A_{Genital}(a-1,i)+\gamma_{Genital}I_{Genital}(a,i)-\left(\eta(t,a)+\mu(a)+\pi_{Genital}\right)A_{Genital}(a,i) \\ \end{matrix}\right.$$

We assumed variation in the risk of oral exposure to HSV-1 infection by age. Otherwise, we assumed no additional behavioral heterogeneity in the oral (non-sexual) risk of exposure to HSV-1 infection.

Meanwhile, we assumed that there is heterogeneity in the genital (sexual) risk of exposure to HSV-1 infection—the risk of exposure varied by age, and for each age group, the population was divided into three sexual risk groups $i$ $\left(i=1,2,3\right)$ representing lower to higher sexual risk behavior.

The index $a$ denotes the age cohort $\left(a=1,2,...,20\right)$ in the population—each age group represents a five-year age band (0–4, 5–9, …, 95–99 years old). The subscripts “Oral” and “Genital” represent the mode of acquisition (portal of entry) for HSV-1 infection.

Definitions of all symbols in the equations can be found in Table S1.

**Table S1.** Definitions of symbols in the model equations.

| Symbol | Definition |
| --- | --- |
| $S(a,i)$ | HSV-1 susceptible population |
| $I_{Oral}(a,i)$ | Infected and infectious populations who acquired the infection orally |
| $I_{Genital}(a,i)$ | Infected and infectious populations who acquired the infection genitally |
| $A_{Oral}(a,i)$ | Latently-infected (non-shedding) populations who acquired the infection orally |
| $A_{Genital}(a,i)$ | Latently-infected (non-shedding) populations who acquired the infection genitally |
| $\gamma_{Oral}$ | Rate of progression from the active to the latent stage for oral infection |
| $\gamma_{Genital}$ | Rate of progression from the active to the latent stage for genital infection |
| $\pi_{Oral}$ | Rate of progression from the latent to the active stage for oral infection |
| $\pi_{Genital}$ | Rate of progression from the latent to the active stage for genital infection |
| $\mu(a)$ | Transition rate from one age group to the next age group |
| $N(i)$ | Total population size of each sexual risk group $i$ |
| $\beta(t)$ | Population growth rate |
| $\eta(t,a)$ | Natural mortality rate |
| $\lambda_{Oral}(a,i)$ | Oral HSV-1 force of infection experienced by each susceptible $S(a,i)$ population |
| $\lambda_{Genital}(a,i)$ | Genital HSV-1 force of infection experienced by each susceptible $S(a,i)$ population |

For each age group and sexual risk group, the population was stratified into five epidemiological categories. $S(a,i)$ represents the HSV-1 susceptible population. $I_{Oral}(a,i)$ and $I_{Genital}(a,i)$ represent the infected and infectious populations (whether for primary infection or reactivations) who acquired the infection orally and genitally, respectively. $A_{Oral}(a,i)$ and $A_{Genital}(a,i)$ represent the latently-infected (non-shedding) populations who acquired the infection orally and genitally, respectively. HSV-1 genital acquisition was assumed to occur only among those who reached their sexual debut (defined as ≥15 years of age; $a>3$). The fraction of HSV-1 genital herpes infections that are symptomatic is denoted by $f_{Symptomatic}^{Genital}$ (not shown in the model equations, but used to calculate the ever-symptomatic genital herpes prevalence).

The natural history of HSV-1 infection was assumed to depend on the mode of acquisition. $\gamma_{Oral}$ and $\gamma_{Genital}$ are the rates of progression from the active (shedding) to the latent (non-shedding) stage for each of oral and genital infections, respectively. $\pi_{Oral}$ and $\pi_{Genital}$ are the rates of progression from the latent to the active stage for each of oral and genital infections, respectively.

$\mu(a)$ is the transition rate from one age group to the next age group. $N(i)$ is the total population size of each sexual risk group $i$. The population growth rate ($\beta(t)$) and the natural mortality rate $\left(\eta(t,a)\right)$ were described by the following functions, as they provided a robust fit of the population growth and age structure in the United States (US) ([1](#_ENREF_1)):

$$\beta(t)=a_{0}e^{-\left(\frac{t-t_{0}}{b}\right)^{2}}$$

and

$$\eta(t,a)=\frac{a_{1}e^{-\left(\frac{t-t_{1}}{b_{1}}\right)^{2}}}{\left[1+e^{-b_{2}\left(a-a_{2}\right)}\right]}$$

Here $a_{0}$, $a_{1}$, $a_{2}$, $t_{0}$, $t_{1}$, $b_{0}$, $b_{1}$, and $b_{2}$ are fitting parameters.

The rates $\lambda_{Oral}(a,i)$ and $\lambda_{Genital}(a,i)$ are the oral and genital HSV-1 forces of infection (hazard rates of infection), respectively, experienced by each susceptible $S(a,i)$ population. The oral HSV-1 force of infection consists of two forces of infection each of which describing a different sub-mode of acquisition, that is oral-to-oral and genital-to-oral (the latter through oral sex):

$$\lambda_{Oral}(a,i)=\lambda_{Oral\text{ }to\text{ }oral\text{ }}(a,i)+\lambda_{\begin{matrix} Genital\text{ }to\text{ }oral \\ \text{ }(oral\text{ }sex) \end{matrix}}(a,i)$$

where

$$\lambda_{Oral\text{ }to\text{ }oral}(a,i)=\rho_{S(a,i)}^{Oral}\sum_{a'=1}^{20} t_{I_{Oral}(a',i)\to S(a,i)}^{Oral}H_{_{a,a'}}^{Oral}\frac{\rho_{I_{Oral}(a',i)}^{Oral}I_{Oral}(a',i)}{\rho_{S(a',i)}^{Oral}S(a',i)+\sum_{\chi=Oral,Genital} \sum_{Z_{\chi}=I_{\chi},A_{\chi}} \rho_{_{Z_{\chi}(a',i)}}^{Oral}Z_{^{\chi}}(a',i)}$$

$$\lambda_{\begin{matrix} Genital\text{ }to\text{ o}ral \\ \text{ }(Oral\text{ }sex) \end{matrix}}(a,i)=\rho_{S(a,i)}^{Oral\text{ }sex}\sum_{a'=1}^{20} \sum_{i'=1}^{3} t_{I_{Genital}(a',i')\to S(a,i)}^{Oral\text{ }sex}H_{_{a,a'}}^{Oral\text{ }sex}G_{_{i,i'}}^{Oral\text{ }sex}\frac{\rho_{I_{Oral}(a',i')}^{Oral\text{ }sex}I_{Genital}(a',i')}{\rho_{S(a',i')}^{Oral\text{ }sex}S(a',i')+\sum_{Z=I_{Oral},A_{Oral}} \rho_{_{Z(a',i')}}^{Oral\text{ }sex}Z(a',i')+\sum_{Z=I_{Genital},A_{Genital}} \rho_{_{Z(a',i')}}^{Oral\text{ }sex}Z(a',i')}$$

The genital HSV-1 force of infection consists of two forces of infection each of which describing a different sub-mode of acquisition, that is oral-to-genital (through oral sex) and genital-to-genital (through sexual intercourse): $\lambda_{Genital}(a,i)=\lambda_{\begin{matrix} Oral\text{ }to\text{ }genital \\ \text{ }(oral\text{ }sex) \end{matrix}}(a,i)+\lambda_{\begin{matrix} Genital to genital \\ (sexual intercourse) \end{matrix}}(a,i)$

where

$$\lambda_{\begin{matrix} Oral\text{ }to\text{ }genital \\ \text{ }(oral\text{ }sex) \end{matrix}}(a,i)=\rho_{S(a,i)}^{Oral\text{ }sex}\sum_{a'=1}^{20} \sum_{i'=1}^{3} t_{I_{Oral}(a',i')\to S(a,i)}^{Oral\text{ }sex}H_{_{a,a'}}^{Oral\text{ }sex}G_{_{i,i'}}^{Oral\text{ }sex}\frac{\rho_{I_{Oral}(a',i')}^{Oral\text{ }sex}I_{Oral}(a',i')}{\rho_{S(a',i')}^{Oral\text{ }sex}S(a',i')+\sum_{Z=I_{Oral},A_{Oral}} \rho_{_{Z(a',i')}}^{Oral\text{ }sex}Z(a',i')+\sum_{Z=I_{Genital},A_{Genital}} \rho_{_{Z(a',i')}}^{Oral\text{ }sex}Z(a',i')}$$

$$\lambda_{\begin{matrix} Genital to genital \\ (sexual intercourse) \end{matrix}}(a,i)=\rho_{S(a,i)}^{Sexual intercourse}\sum_{a'=1}^{20} \sum_{i'=1}^{3} t_{I_{Genital}(a',i')\to S(a,i)}^{Sexual intercourse}H_{_{a,a'}}^{Sexual intercourse}G_{_{i,i'}}^{Sexual intercourse}\frac{\rho_{I_{Genital}(a',i')}^{Sexual intercourse}I_{Genital}(a',i')}{\rho_{S(a',i')}^{Sexual intercourse}S(a',i')+\sum_{Z=I_{Oral},A_{Oral}} \rho_{_{Z(a',i')}}^{Sexual intercourse}Z(a',i')+\sum_{Z=I_{Genital},A_{Genital}} \rho_{_{Z(a',i')}}^{Sexual intercourse}Z(a',i')}$$

Here, $\rho_{X(a,i)}^{Oral}$ is the effective non-sexual oral infectious contact rate per year, while $\rho_{X(a,i)}^{Oral\text{ sex}}$ and $\rho_{X(a,i)}^{Sexual intercourse}$ describe the effective sexual partner change rates for oral sex and for sexual intercourse, respectively, per year for each population variable $X(a,i)$. This formulation of the force of infection assumes implicitly that an oral sex partnership is reciprocal by nature—both partners engage in active and receptive roles.

We assumed that there is no HSV-1 transmission during the latent stages for lack of viral shedding. The parameter $t_{I_{Oral}(a',i')\to S(a,i)}^{Oral}$ describes the oral non-sexual HSV-1 transmission probability per “partnership” between a member of the susceptible population $S(a,i)$ and a member of the infected population $I_{Oral}(a,i)$. This parameter $\left(t_{I_{Oral}(a',i')\to S(a,i)}^{Oral}\right)$ and the effective non-sexual oral infectious contact rate per year $\left(\rho_{X(a,i)}^{Oral}\right)$ were combined into one fitting parameter, due to absence of data determining them.

The parameters $t_{I_{Oral}(a',i')\to S(a,i)}^{Oral\text{ }sex}$ and $t_{I_{Genital}(a',i')\to S(a,i)}^{Oral\text{ }sex}$ describe the oral sex HSV-1 transmission probability per sexual partnership between a member of the susceptible population $S(a,i)$ and a member of the infected population $I_{Oral}(a,i)$ and $I_{Genital}(a,i)$, respectively:

$$t_{I_{Oral}(a',i')\to S(a,i)}^{Oral\text{ }sex}=t_{I_{Genital}(a',i')\to S(a,i)}^{Oral\text{ }sex}=1-\left(1-p_{I_{Oral}(a',i')\to S(a,i)}\right)^{n_{I_{Oral}(a',i')\leftrightarrow S(a,i)}}$$

The oral sex HSV-1 transmission probability per partnership is expressed in terms of HSV-1 transmission probability per sex act $\left(p_{I_{Oral}(a',i')\to S(a,i)}\right)$, and the frequency of sex acts per year in this partnership $\left(n_{I_{Oral}(a',i')\leftrightarrow S(a,i)}\right)$.

The parameter $t_{I_{Genital}(a',i')\to S(a,i)}^{Sexual intercourse}$ describes the sexual intercourse HSV-1 transmission probability, per sexual partnership between a member of the susceptible population $S(a,i)$ and a member of the infected population $I_{Genital}(a,i)$:

$$t_{I_{Genital}(a',i')\to S(a,i)}^{Sexual intercourse}=1-\left(1-p_{I_{Genital}(a',i')\to S(a,i)}\right)^{n_{I_{Genital}(a',i')\leftrightarrow S(a,i)}}$$

The sexual intercourse HSV-1 transmission probability per partnership is expressed in terms of HSV-1 transmission probability per sex act $\left(p_{I_{Genital}(a',i')\to S(a,i)}\right)$, and the frequency of sex acts per year in this partnership $\left(n_{I_{Genital}(a',i')\leftrightarrow S(a,i)}\right)$.

The mixing among different age groups is dictated by the mixing matrices $H_{_{a,a'}}^{Oral}$, $H_{_{a,a'}}^{Oral\text{ }sex}$, and $H_{_{a,a'}}^{Sexual intercourse}$. The mixing among the different sexual risk groups is dictated by the mixing matrices $G_{_{i,j}}^{Oral\text{ }sex}$ and $G_{_{i,j}}^{Sexual intercourse}$. These matrices provide the likelihood of mixing and are given by the following expressions:

$$H_{_{a,a'}}^{Oral}=e_{Age}^{Oral}\delta_{a,a'}+(1-e_{Age}^{Oral})\frac{\sum_{i=1}^{3} \left(\rho_{S(a',i')}^{Oral}S(a',i)+\sum_{Z=I_{Oral},A_{Oral}} \rho_{_{Z(a',i)}}^{Oral}Z(a',i)+\sum_{Z=I_{Genital},A_{Genital}} \rho_{_{Z(a',i)}}^{Oral}Z(a',i)\right)}{\sum_{a''=1}^{20} \sum_{i=1}^{3} \left(\rho_{S(a'',i')}^{Oral}S(a'',i)+\sum_{Z=I_{Oral},A_{Oral}} \rho_{_{Z(a'',i)}}^{Oral}Z(a'',i)+\sum_{Z=I_{Genital},A_{Genital}} \rho_{_{Z(a'',i)}}^{Oral}Z(a'',i)\right)}$$

$$H_{_{a,a'}}^{Oral\text{ }sex}=e_{Age}^{Oral\text{ }sex}\delta_{a,a'}+(1-e_{Age}^{Oral\text{ }sex})\frac{\sum_{i=1}^{3} \left(\rho_{S(a',i')}^{Oral\text{ }sex}S(a',i)+\sum_{Z=I_{Oral},A_{Oral}} \rho_{_{Z(a',i)}}^{Oral\text{ }sex}Z(a',i)+\sum_{Z=I_{Genital},A_{Genital}} \rho_{_{Z(a',i)}}^{Oral\text{ }sex}Z(a',i)\right)}{\sum_{a''=1}^{20} \sum_{i=1}^{3} \left(\rho_{S(a'',i')}^{Oral\text{ }sex}S(a'',i)+\sum_{Z=I_{Oral},A_{Oral}} \rho_{_{Z(a'',i)}}^{Oral\text{ }sex}Z(a'',i)+\sum_{Z=I_{Genital},A_{Genital}} \rho_{_{Z(a'',i)}}^{Oral\text{ }sex}Z(a'',i)\right)}$$

$$H_{_{a,a'}}^{Sexual intercourse}=e_{Age}^{Sexual intercourse}\delta_{a,a'}+(1-e_{Age}^{Sexual intercourse})\frac{\sum_{i=1}^{3} \left(\rho_{S(a',i')}^{Sexual intercourse}S(a',i)+\sum_{Z=I_{Oral},A_{Oral}} \rho_{_{Z(a',i)}}^{Sexual intercourse}Z(a',i)+\sum_{Z=I_{Genital},A_{Genital}} \rho_{_{Z(a',i)}}^{Sexual intercourse}Z(a',i)\right)}{\sum_{a''=1}^{20} \sum_{i=1}^{3} \left(\rho_{S(a'',i')}^{Sexual intercourse}S(a'',i)+\sum_{Z=I_{Oral},A_{Oral}} \rho_{_{Z(a'',i)}}^{Sexual intercourse}Z(a'',i)+\sum_{Z=I_{Genital},A_{Genital}} \rho_{_{Z(a'',i)}}^{Sexual intercourse}Z(a'',i)\right)}$$

$$G_{_{i,j}}^{Oral\text{ }sex}=e_{Risk}^{Oral\text{ }sex}\delta_{i,j}+(1-e_{Risk}^{Oral\text{ }sex})\frac{\sum_{a=1}^{20} \left(\rho_{S(a,i')}^{Oral\text{ }sex}S(a,j)+\sum_{Z=I_{Oral},A_{Oral}} \rho_{_{Z(a,i')}}^{Oral\text{ }sex}Z(a,j)+\sum_{Z=I_{Genital},A_{Genital}} \rho_{_{Z(a,i')}}^{Oral\text{ }sex}Z(a,j)\right)}{\sum_{a=1}^{20} \sum_{i'=1}^{3} \left(\rho_{S(a,i')}^{Oral\text{ }sex}S(a,i')+\sum_{Z=I_{Oral},A_{Oral}} \rho_{_{Z(a,i')}}^{Oral\text{ }sex}Z(a,i')+\sum_{Z=I_{Genital},A_{Genital}} \rho_{_{Z(a,i')}}^{Oral\text{ }sex}Z(a,i')\right)}$$

$$G_{_{i,j}}^{Sexual intercourse}=e_{Risk}^{Sexual intercourse}\delta_{i,j}+(1-e_{Risk}^{Sexual intercourse})\frac{\sum_{a=1}^{20} \left(\rho_{S(a,i')}^{Sexual intercourse}S(a,j)+\sum_{Z=I_{Oral},A_{Oral}} \rho_{_{Z(a,i')}}^{Sexual intercourse}Z(a,j)+\sum_{Z=I_{Genital},A_{Genital}} \rho_{_{Z(a,i')}}^{Sexual intercourse}Z(a,j)\right)}{\sum_{a=1}^{20} \sum_{i'=1}^{3} \left(\rho_{S(a,i')}^{Sexual intercourse}S(a,i')+\sum_{Z=I_{Oral},A_{Oral}} \rho_{_{Z(a,i')}}^{Sexual intercourse}Z(a,i')+\sum_{Z=I_{Genital},A_{Genital}} \rho_{_{Z(a,i')}}^{Sexual intercourse}Z(a,i')\right)}$$

Here, $\delta_{a,a'}$ (and$\delta_{i,j}$) is the identity matrix.$e_{Age}^{Oral}$, $e_{Age}^{Oral\text{ }sex}$, $e_{Age}^{Sexual intercourse}$, $e_{Risk}^{Oral\text{ }sex}$ and $e_{Risk}^{Sexual intercourse}\in\left[0,1\right]$ measure the degree of assortativeness in the mixing. At the extreme $e_{Age}^{Oral}=e_{Age}^{Oral\text{ }sex}=e_{Age}^{Sexual intercourse}=e_{Risk}^{Oral\text{ }sex}=e_{Risk}^{Sexual intercourse}=0$ , the mixing is fully proportional while at the other extreme $e_{Age}^{Oral}=e_{Age}^{Oral\text{ }sex}=e_{Age}^{Sexual intercourse}=e_{Risk}^{Oral\text{ }sex}=e_{Risk}^{Sexual intercourse}=1$, the mixing is fully assortative, that is individuals mix only with members in their own risk or age group.

- 1. *Non-sexual oral contact rate*

To accommodate for temporal variation in HSV-1 seroprevalence, the model was developed to include temporal variation in the risk of exposure through the oral-to-oral mode of acquisition—thereby accommodating the changes in hygiene and socioeconomic conditions in the US in recent decades ([2-5](#_ENREF_2)). We parameterized this variation (time dependence of $\rho_{X(a,i)}^{Oral}$) through a Wood-Saxon function ([6](#_ENREF_6), [7](#_ENREF_7)). This function is mathematically designed to describe and characterize transitions in terms of their scale or strength, smoothness or abruptness, thickness (duration), and the turning point ([6-8](#_ENREF_6)). Using the Wood-Saxon parameterization, $\rho_{X(a,i)}^{Oral}$ is given by:

$$\rho_{X(\mathrm{a},i)}^{Oral}(t)=\rho_{X(\mathrm{a},i)}^{Oral}\times\left(1+\frac{Z}{1+\exp\left[\left(t-\xi_{Turning}\right)/\xi_{Duration}\right]}\right)$$

Here, $\rho_{X(\mathrm{a},i)}^{Oral}$ is the asymptotic value of $\rho_{X(\mathrm{a},i)}^{Oral}(t)$ that describes the level of risk of exposure well after the transition, and $\xi_{Duration}$ describes the transition duration parameter. Meanwhile, $\xi_{Turning}$ is the turning point year at which the rate of contacts per year crosses half the way towards its asymptotic value of $\rho_{X(\mathrm{a},i)}^{Oral}$.

The level of risk of exposure changes during the transition from $\rho_{X(a,i)}^{Oral}\times\left(1+Z\right)$ before the transition to $\rho_{X(a,i)}^{Oral}$ after the transition. Accordingly, the reduction in the risk of exposure is given by .

- 1. *Sexual contact rates*

The parameters $\rho_{X(a,i)}^{Oral\text{ }sex}$ and $\rho_{X(a,i)}^{Sexual intercourse}$ describe the effective sexual partner change rates per year for each of oral sex and sexual intercourse, respectively, for each population variable. These parameters describe effectively a composite of behavioral factors that enhance the sexual risk of exposure to the infection such as variability in risk of exposure, concurrency, and clustering within sexual networks ([9-12](#_ENREF_9)).

The $\rho_{X(a,i)}^{Oral\text{ }sex}$ and $\rho_{X(a,i)}^{Sexual intercourse}$ distributions across the different risk groups and age groups were defined through a power law function:

$$\rho_{X(a,i)}^{\omega}(a,i)=C^{\omega}\times\psi(a)\times i^{\theta}$$

This function was motivated by analyses of the architecture of complex weighted networks ([13](#_ENREF_13), [14](#_ENREF_14)), and by an analysis of the average separation between individuals in a network or a sub-network ([15](#_ENREF_15)). Here, $C^{\omega}$ ($\omega$ standing for each of oral sex and sexual intercourse) is a constant determined by the average risk of exposure and $\theta$ is the exponent parameter that determines the growth in risk of exposure with risk group number $i$. To account for the variability in sexual activity by age, we incorporated the parameter $\psi(a)$ that describes the variation by age, as determined using the sexual partnership data of the 2013-2014 National Health and Nutrition Examination Survey (NHANES) ([16](#_ENREF_16)).

No temporal variation in the sexual partnership change rates was assumed.

**Supplemental Information 2: Parameter values**

- 1. *Input parameters*

The parameters of the model were derived using current empirical data on HSV-1 natural history and epidemiology, and are listed in Table S1 along with their references. The HSV-1 transmission probability per oral sex act or sexual intercourse act was assumed to be equal to that of HSV-2 for sexual intercourse ([17](#_ENREF_17), [18](#_ENREF_18)). This assumption, however, is of no consequence for the case of the oral sex component, as any difference in the transmission probability is effectively captured (through model fitting) by the partner change rates.

Oral HSV-1 shedding frequency was assumed to occur 13.7% of the time, while genital HSV-1 shedding frequency was assumed to occur 1.5% of the time. These parameters were based on direct measurement from prospective cohort studies ([19-21](#_ENREF_19)). Moreover, oral and genital HSV-1 shedding was assumed to occur through $\upsilon_{Oral}=16.2$ and $\upsilon_{Genital}=0.7$ reactivations per year, with each complete oral and genital cycle (of reactivation and latency) lasting for $\tau_{Cycle}^{Oral}=\left(365/\upsilon_{Oral}\right)=22.5$ days and $\tau_{Cycle}^{Genital}=\left(365/\upsilon_{Genital}\right)=521.4$ days, respectively. Within the cycle, the duration of latency between two reactivations is given by

$$\frac{1}{\pi_{Oral}}=\left(\frac{1-\xi_{Oral}}{\upsilon_{Oral}}\right)\times365$$

and

$$\frac{1}{\pi_{Genital}}=\left(\frac{1-\xi_{Genital}}{\upsilon_{Genital}}\right)\times365$$

Meanwhile, the duration of reactivation within the cycle is provided by

$$\frac{1}{\gamma_{Oral}}=\left(\frac{\xi_{Oral}}{\upsilon_{Oral}}\right)\times365$$

and

$\frac{1}{\gamma_{Genital}}=\left(\frac{\xi_{Genital}}{\upsilon_{Genital}}\right)\times365$.

We assumed that primary HSV-1 infection has the same duration as that of reactivation.

The proportion of the population in each sexual risk group $i$ was calculated based on NHANES data for the distribution of sexual partnerships over the past 12 months ([16](#_ENREF_16)). The degree of assortativeness ($e_{Risk}^{Oral\text{ }sex}$ and $e_{Risk}^{Sexual intercourse}$) for sexual risk group mixing was fixed at 0.3, and the degree of assortativeness ($e_{Age}^{Oral\text{ }sex}$ and $e_{Age}^{Sexual intercourse}$) for age group mixing was fixed at 0.7, for both oral sex and sexual intercourse partnerships,—representative values informed by earlier modeling work ([9](#_ENREF_9), [22](#_ENREF_22)). The frequency of sex acts per age group for both oral sex and sexual intercourse partnerships was based on the measurements of Weinstein *et al.* ([23](#_ENREF_23)).

The parameters of the model that were derived from the existing literature are listed in Table S2, along with their references.

**Table S2.** Model assumptions in terms of parameter values.

| **Parameter** | **Symbol** | **Value** | **Range for uncertainty interval (**±**30%)** | **Justification** | **Sources** |
| --- | --- | --- | --- | --- | --- |
| HSV-1 transmission probability per act for an oral sex act | $p_{I_{Oral}(a',i')\to S(a,i)}$ | 0.01 | 0.007-0.013 | Assumed equal to that of HSV-2 for sexual intercourse. This assumption is of no consequence as any difference in the transmission probability is effectively captured (through model fitting) by the partner change rates | ([17](#_ENREF_17), [18](#_ENREF_18)) |
| HSV-1 transmission probability per act for a sexual intercourse act | $p_{I_{Genital}(a',i')\to S(a,i)}$ | 0.01 | 0.007-0.013 | Assumed equal to that of HSV-2 for sexual intercourse | ([17](#_ENREF_17), [18](#_ENREF_18)) |
| Oral HSV-1 shedding frequency | $\xi_{Oral}$ | 13.7% of the time | 9.59-17.81% of the time | Direct measurement from a prospective cohort study | ([19](#_ENREF_19))(1) |
| Genital HSV-1 shedding frequency | $\xi_{Genital}$ | 1.5% of the time | 1.05-1.95% of the time | Direct measurement from a prospective cohort study | ([21](#_ENREF_21)) |
| Frequency of oral HSV-1 reactivations | $\chi_{Oral}$ | 16.2 per year | 11.34-21.06 per year | Direct measurement from a prospective cohort study | ([21](#_ENREF_21)) |
| Frequency of genital HSV-1 reactivations | $\chi_{Genital}$ | 0.7 per year | 0.49-0.91 per year | Direct measurement from a prospective cohort study | ([20](#_ENREF_20)) |
| Duration of latency between two oral reactivations | $\frac{1}{\pi_{Oral}}$ | 19.4 days | 13.58-25.22 days | Derived (see text above) | ([17](#_ENREF_17)) |
| Duration of latency between two genital reactivations | $\frac{1}{\pi_{Genital}}$ | 513.5 days | 359.45-667.55 days | Derived (see text above) | ([17](#_ENREF_17)) |
| Duration of an oral reactivation | $\frac{1}{\gamma_{Oral}}$ | 3.1 days | 2.17-4.03 days | Derived (see text above) | ([17](#_ENREF_17)) |
| Duration of a genital reactivation | $\frac{1}{\gamma_{Genital}}$ | 7.9 days | 5.53-10.27 days | Derived (see text above) | ([17](#_ENREF_17)) |
| Degree of sexual assortativeness for age group mixing | $e_{Age}^{Genital}$ | 0.7 | 0.49-0.91 | Informed by earlier modeling work | ([22](#_ENREF_22)) |
| Degree of sexual assortativeness for sexual risk group mixing | $e_{_{Risk}}^{Genital}$ | 0.3 | 0.21-0.39 | Informed by earlier modeling work | ([9](#_ENREF_9)) |
| Fraction of HSV-1 genital herpes infections that are symptomatic | $f_{Symptomatic}^{Genital}$ | 26% | 18.2-33.8% | Direct measurement from a prospective cohort study | ([24](#_ENREF_24)) |

- 1. *Fitting parameters derived by fitting empirical data in this study*

The constant $C^{Sexual intercourse}$ was determined by adapting an earlier model for HSV-2 transmission ([17](#_ENREF_17)) and fitting it to NHANES data for HSV-2 infection ([16](#_ENREF_16)), to derive the overall level of sexual-intercourse risk behavior in the US population. The age group mixing for the oral mode of transmission ($e_{1}^{Oral}$), the exponent parameter ($\theta$), the constants $C^{Oral\text{ }sex}$, $\rho_{X(a,i)}^{Oral}$, $Z$, $\xi_{Turning}$, and $\xi_{Duration}$ were determined by fitting HSV-1 seroprevalence over time and age groups, as well as HSV-1 genital herpes prevalence among HSV-1 positive individuals, both per the different rounds of NHANES data ([16](#_ENREF_16)).

**Supplemental Information 3: A** **heuristic explanation of HSV-1 genital herpes 50% seroprevalence turning point**

The intriguing growth but then saturation of genital herpes contribution was found to reflect the subtle dynamics of the oral-to-genital sexual mode of transmission. HSV-1 oral-to-genital incidence (through oral sex) was found to be driven not only by the increasingly larger susceptible population reaching sexual debut uninfected, but critically by the *large reservoir* of orally-acquired HSV-1 infections. This can be seen (heuristically) as expressed mathematically (noting that $1-Seroprevalence_{HSV-1}$ is the susceptible fraction of the population):

$$Incidence_{oral-to-genital}\propto\left(1-Seroprevalence_{HSV-1}\right)\times Prevalence_{Oral\text{ }herpes}\approx\left(1-Seroprevalence_{HSV-1}\right)\times Seroprevalence_{HSV-1}$$

Accordingly, as seroprevalence declines from its high levels, the term $\left(1-Seroprevalence_{HSV-1}\right)\times Seroprevalence_{HSV-1}$ increases reaching its peak at 50% seroprevalence and declining thereafter (Fig. S2).

**Fig S2. The dynamics of the oral-to-genital sexual mode of transmission and the HSV-1 epidemiology turning point.** The curve below shows the variation of the function: $\left(1-Seroprevalence_{HSV-1}\right)\times Seroprevalence_{HSV-1}$. This function illustrates how the contribution of the oral-to-genital sexual mode of transmission *increases* as HSV-1 seroprevalence declines down to 50% seroprevalence, after which the contribution *decreases* with further declines in seroprevalence.


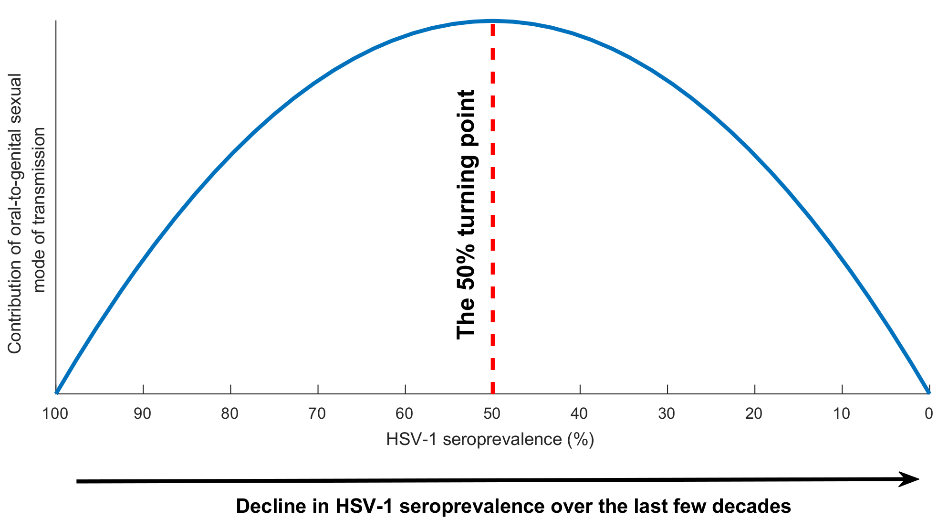


This is the essence of what has been unfolding in the US in recent decades. HSV-1 seroprevalence was historically at very high level, but was declining steadily for several decades. As seroprevalence approached the 50% *turning point*, the contribution of genital herpes to incidence peaked leading to HSV-1 becoming observed as the leading cause of first-episode genital herpes ([2](#_ENREF_2), [25](#_ENREF_25)). It is of no surprise then to witness a growing volume of literature indicating increasing incidence of HSV-1 genital herpes, particularly among young adults ([24-26](#_ENREF_24)). As seroprevalence drops below the 50% turning point over the coming decades, this trend will be self-limiting and HSV-1 genital herpes incidence will start to decline (Fig. 4B and Fig. 6C of main text).

In summary, the declining seroprevalence over the past decades has been leading to a larger susceptible young population, and thus *growing* genital herpes incidence, thanks to the large pool of orally-acquired prevalent infections. Eventually, this same declining seroprevalence, will yield (to the contrary) to *declining* genital herpes incidence as the pool of orally-acquired prevalent infections shrinks to an extent that it can no longer sustain as much oral-to-genital transmission through oral sex.

**Fig S3.** **United States demographics.** Estimated population size in the United States (US), compared to the United Nations’ World Population Prospects projection ([1](#_ENREF_1)).


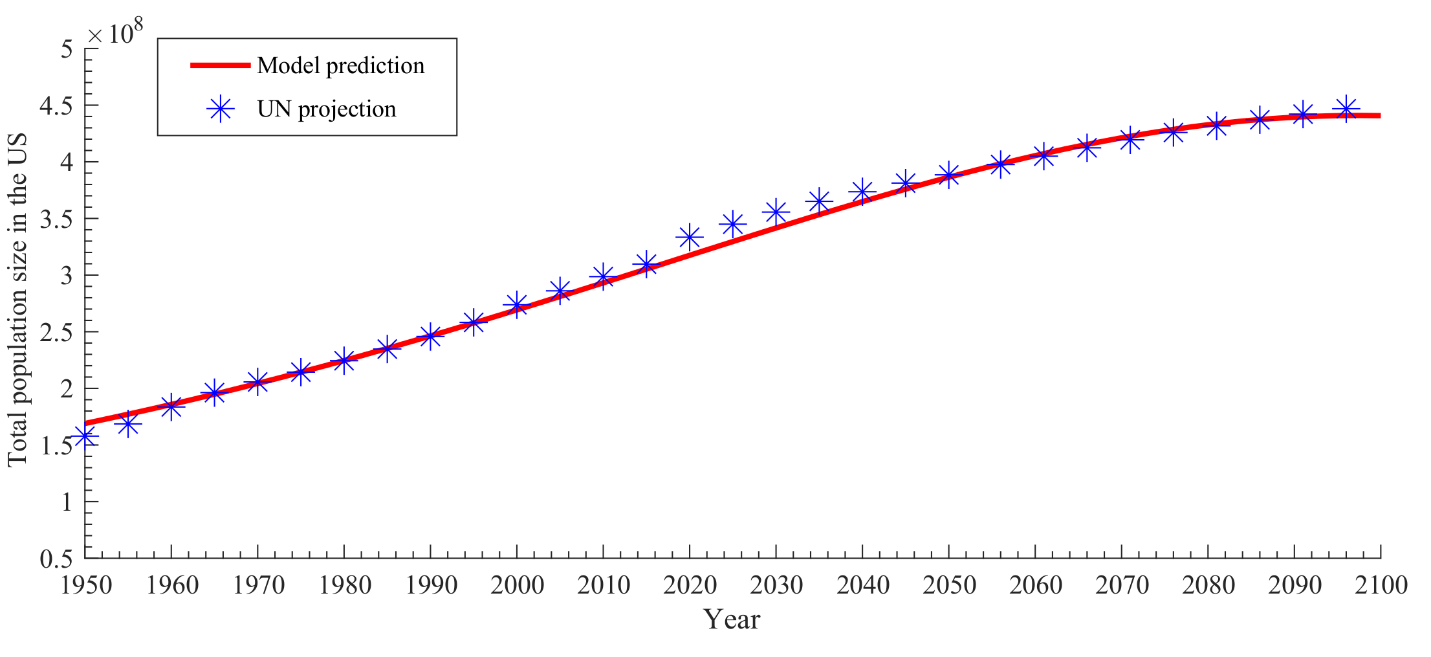


**Fig S4. Temporal evolution of key epidemiologic indicators of HSV-1 infection in adults 15–59 years of age in the United States.** A) Estimated orally-acquired HSV-1 seroprevalence versus genitally-acquired HSV-1 seroprevalence. B) Estimated orally-acquired HSV-1 incidence rate versus genitally-acquired HSV-1 incidence rate. C) Estimated annual number of new (incident) orally-acquired versus genitally-acquired HSV-1 infections.

**
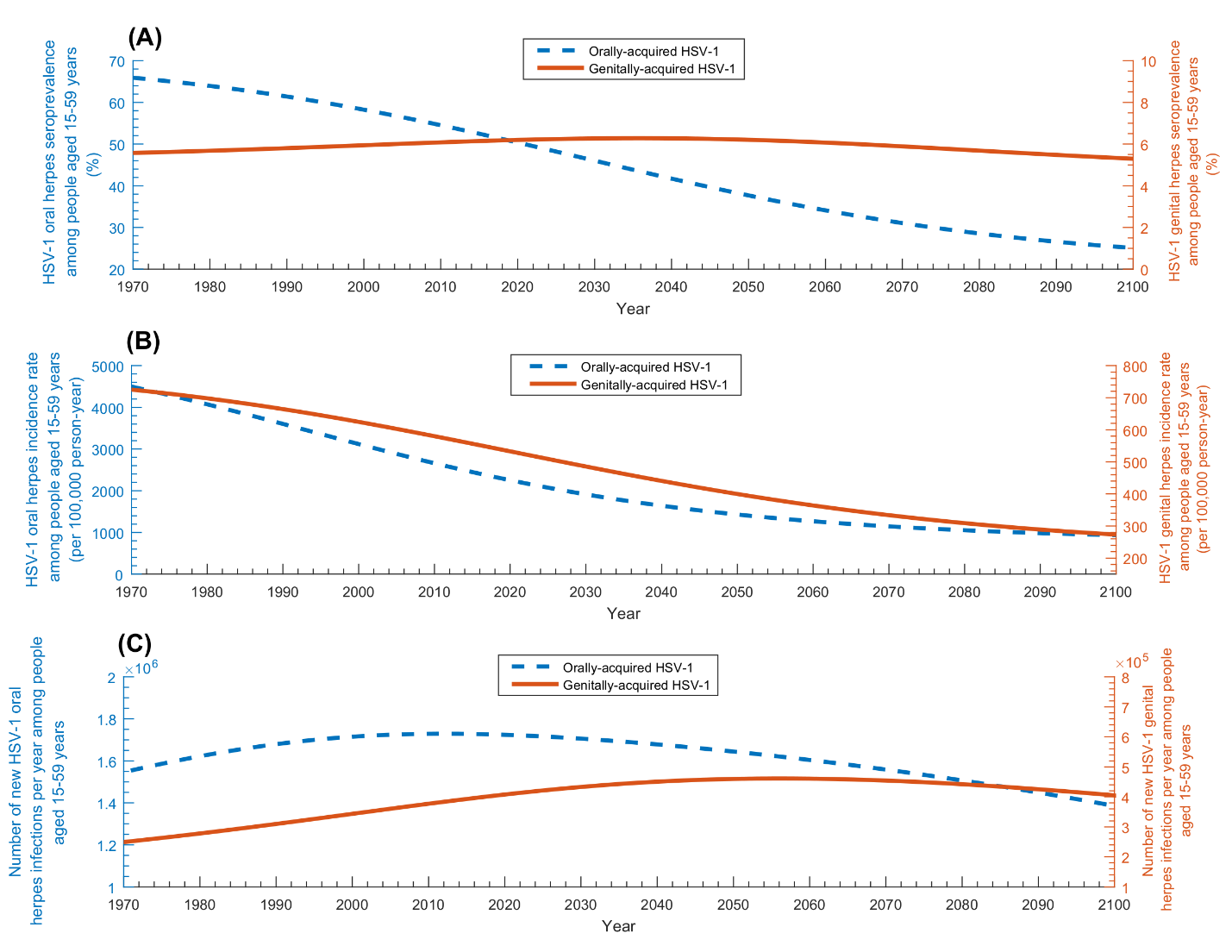
**

**Fig S5. Uncertainty analysis.** Mean and 95% uncertainty interval (UI) for the time trend of the relative contribution of orally-acquired (A) versus genitally-acquired (B) HSV-1 among new (incident) infections in the total population of the United States, and the estimated annual number of new (incident) orally-acquired (C) versus genitally-acquired (D) HSV-1 infections.


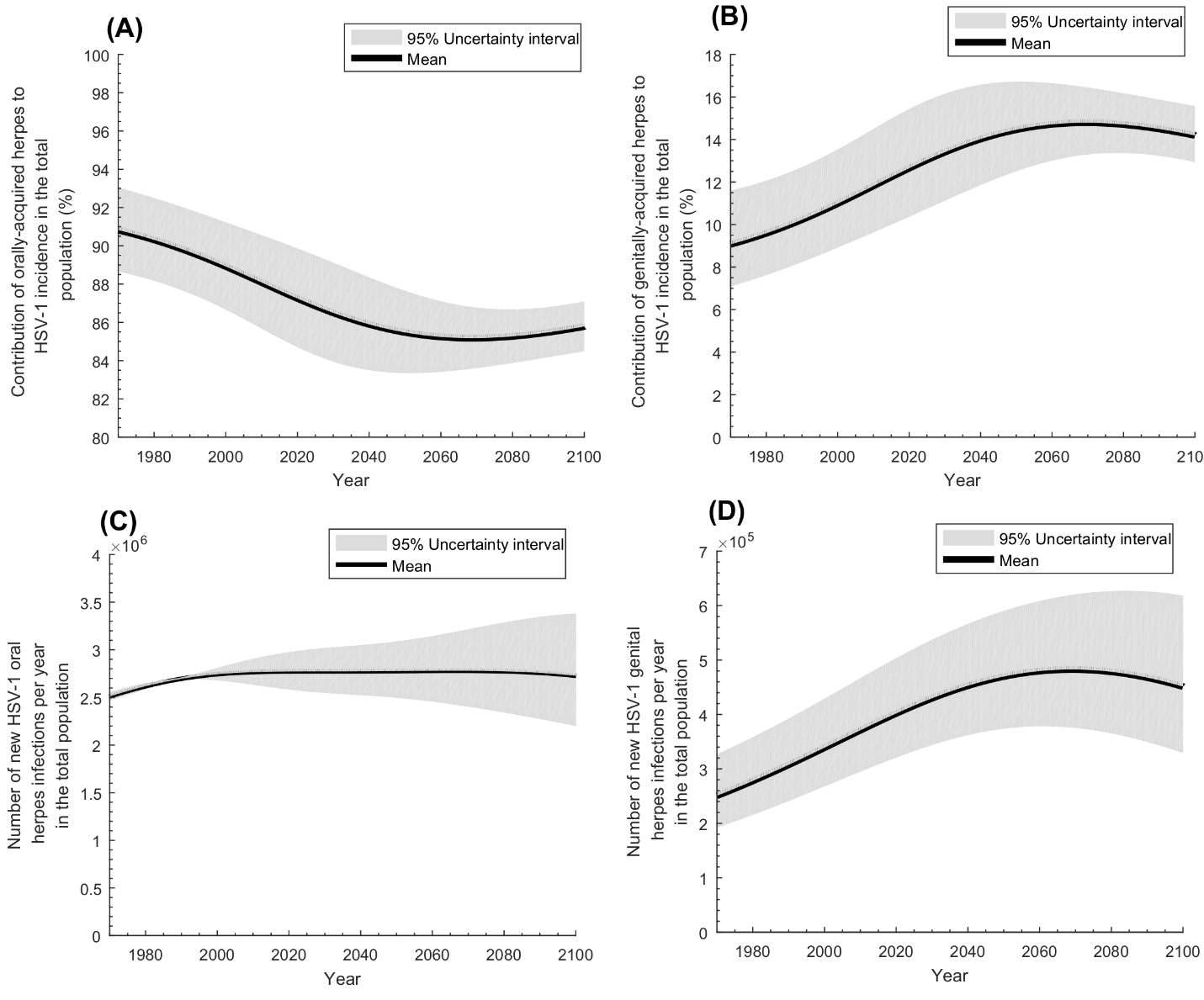


**Fig S6. Sensitivity of model predictions to a wide variation in the oral HSV-1 shedding frequency.** This sensitivity analysis assessed the impact of varying the oral shedding frequency between 5-18%. This range is based on the reported oral shedding frequency in existing studies ([21](#_ENREF_21), [27](#_ENREF_27)). Meanwhile, the baseline model prediction is based on the most current data ([19](#_ENREF_19)). The impact of this wide variation was assessed for the time trend of the relative contribution of orally-acquired (A) versus genitally-acquired (B) HSV-1 among new (incident) infections in the total population of the United States, and the estimated annual number of new (incident) orally-acquired (C) versus genitally-acquired (D) HSV-1 infections.


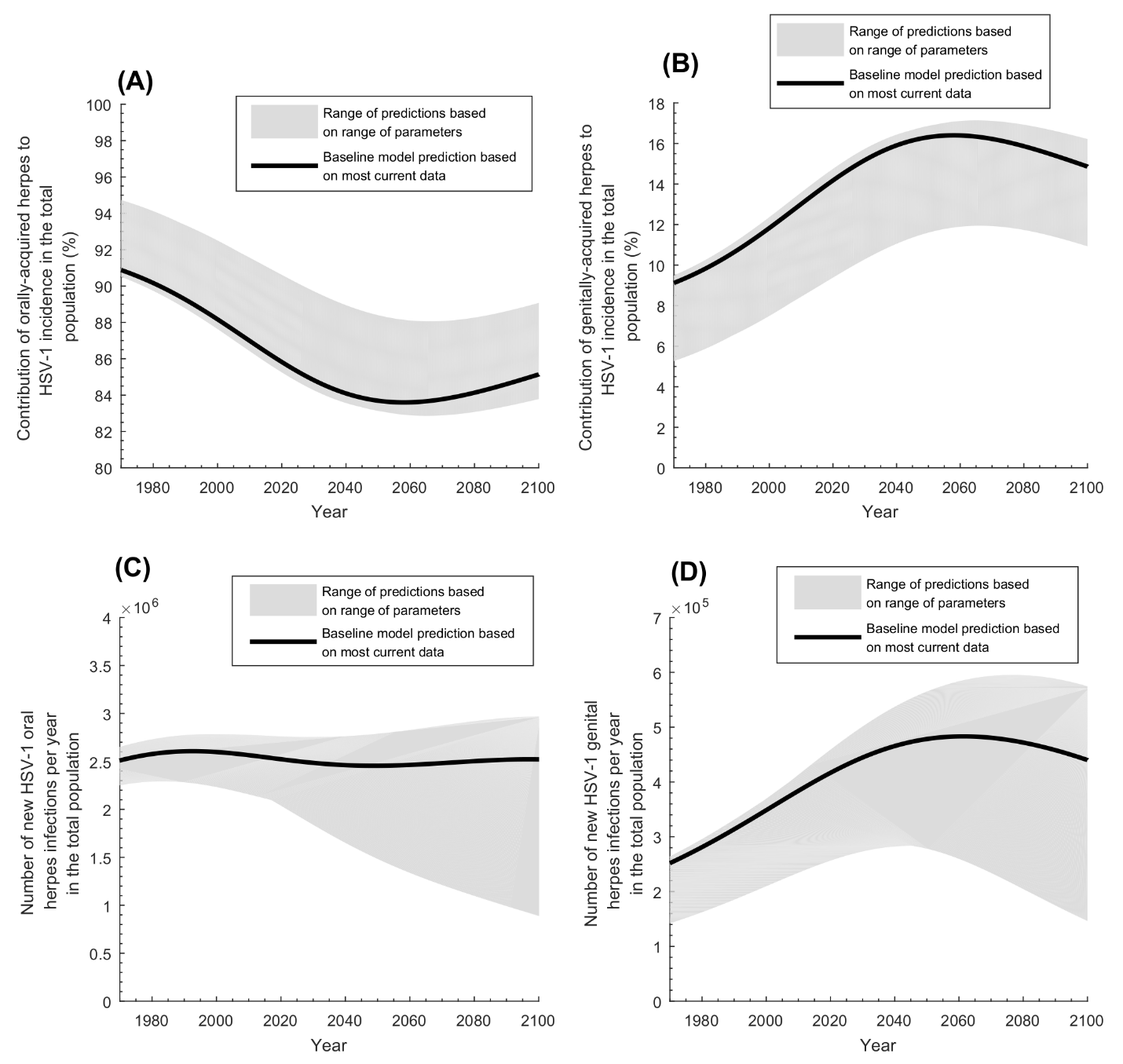


**Fig S7. Sensitivity of model predictions to a wide variation in the genital HSV-1 shedding frequency.** This sensitivity analysis assessed the impact of varying the genital shedding frequency between 0.5-6%. This range is informed by recent unpublished data ([28](#_ENREF_28)), while the baseline model prediction is based on available published evidence ([21](#_ENREF_21)). The impact of this wide variation was assessed for the time trend of the relative contribution of orally-acquired (A) versus genitally-acquired (B) HSV-1 among new (incident) infections in the total population of the United States, and the estimated annual number of new (incident) orally-acquired (C) versus genitally-acquired (D) HSV-1 infections.

*
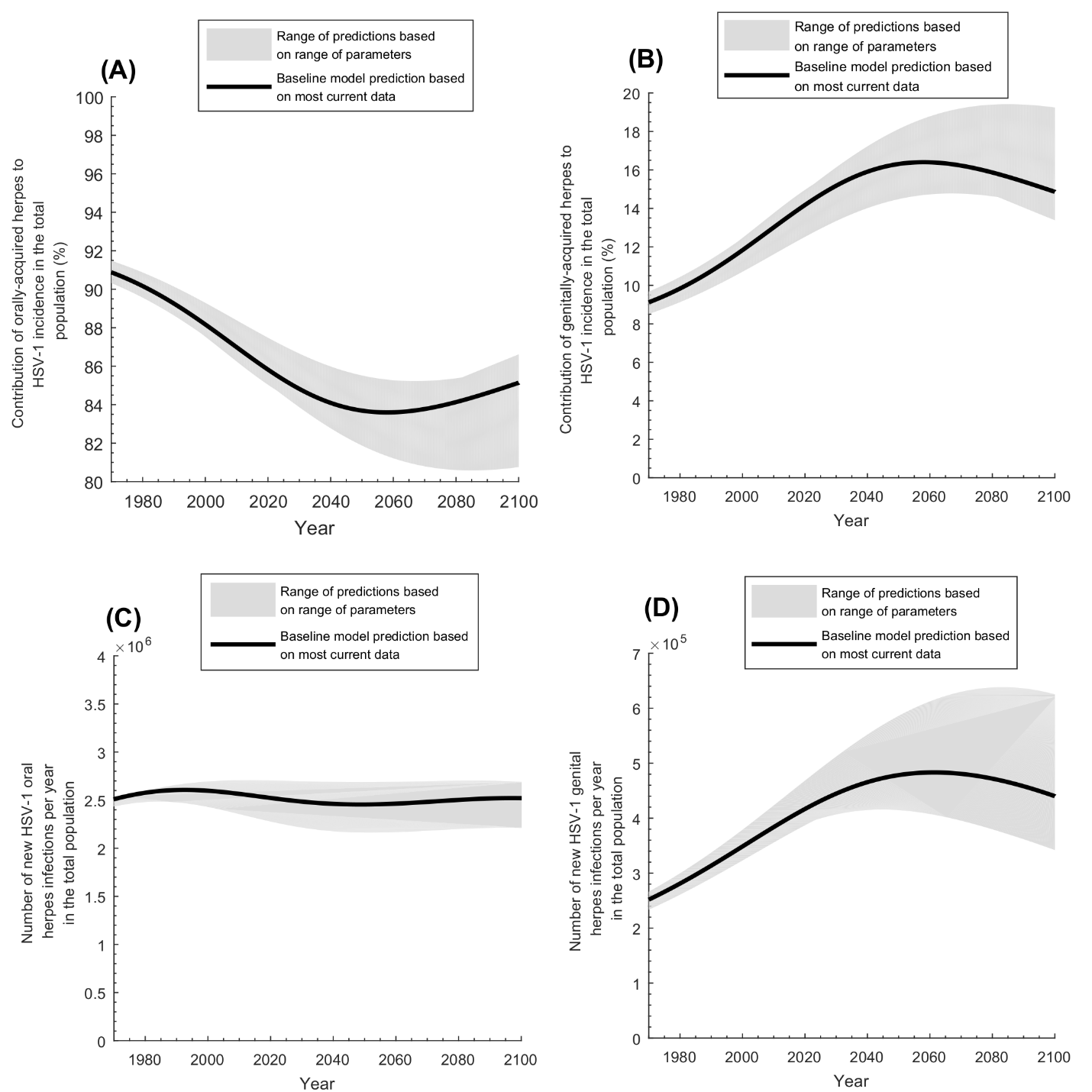
*

**References**

1. Affairs. UNDoEaS. World Population Prospects, the 2015 Revision. <http://esa.un.org/unpd/wpp/>. 2015.

2. Xu F, Sternberg MR, Kottiri BJ, McQuillan GM, Lee FK, Nahmias AJ, et al. Trends in herpes simplex virus type 1 and type 2 seroprevalence in the United States. Jama. 2006;296(8):964-73.

3. Xu F, Lee FK, Morrow RA, Sternberg MR, Luther KE, Dubin G, et al. Seroprevalence of herpes simplex virus type 1 in children in the United States. J Pediatr. 2007;151(4):374-7.

4. Kramer MA, Uitenbroek DG, Ujcic-Voortman JK, Pfrommer C, Spaargaren J, Coutinho RA, et al. Ethnic differences in HSV1 and HSV2 seroprevalence in Amsterdam, the Netherlands. Euro Surveill. 2008;13(24).

5. Sauerbrei A, Schmitt S, Scheper T, Brandstadt A, Saschenbrecker S, Motz M, et al. Seroprevalence of herpes simplex virus type 1 and type 2 in Thuringia, Germany, 1999 to 2006. Euro Surveill. 2011;16(44).

6. Woods RD, Saxon DS. Diffuse surface optical model for nucleon-nuclei scattering. Physical Review. 1954;95(2):577.

7. Velicia FF. On the moments of a (WS) β distribution. Journal of Physics A: Mathematical and General. 1987;20(9):2293.

8. Awad SF, Abu-Raddad LJ. Could there have been substantial declines in sexual risk behavior across sub-Saharan Africa in the mid-1990s? Epidemics. 2014;8:9-17.

9. Abu-Raddad LJ, Longini Jr IM. No HIV stage is dominant in driving the HIV epidemic in sub-Saharan Africa. Aids. 2008;22(9):1055-61.

10. Morris M. Sexual networks and HIV. AIDS (London, England). 1996;11:S209-16.

11. Watts CH, May RM. The influence of concurrent partnerships on the dynamics of HIV/AIDS. Mathematical biosciences. 1992;108(1):89-104.

12. May RM, Anderson RM, Irwin M. The transmission dynamics of human immunodeficiency virus (HIV)[and discussion]. Philosophical Transactions of the Royal Society of London B: Biological Sciences. 1988;321(1207):565-607.

13. Barendregt JJ, Van Oortmarssen GJ, Vos T, Murray CJ. A generic model for the assessment of disease epidemiology: the computational basis of DisMod II. Popul Health Metr. 2003;1(1):4.

14. Barrat A, Barthelemy M, Pastor-Satorras R, Vespignani A. The architecture of complex weighted networks. Proceedings of the National Academy of Sciences of the United States of America. 2004;101(11):3747-52.

15. Barabasi A-L. Linked: How everything is connected to everything else and what it means. Plume Editors. 2002.

16. NHANES. National Health and Nutrition Examination Survey. <http://www.cdc.gov/nchs/nhanes/nhanes_questionnaires.htm>; 1976-2016

17. Abu-Raddad LJ, Magaret AS, Celum C, Wald A, Longini Jr IM, Self SG, et al. Genital herpes has played a more important role than any other sexually transmitted infection in driving HIV prevalence in Africa. PloS one. 2008;3(5):e2230.

18. STD Statistics. <https://srconstantin.wordpress.com/2015/04/30/std-statistics/>.

19. Ramchandani M, Kong M, Tronstein B, Selke S, Mikhaylova A, Huang M-L, et al. Herpes Simplex Virus Type 1 in Tears, and Nasal and Oral Mucosa of Healthy Adults. Sexually Transmitted Diseases. 2016;43(12):756-60.

20. Engelberg R, Carrell D, Krantz E, Corey L, Wald A. Natural history of genital herpes simplex virus type 1 infection. Sexually transmitted diseases. 2003;30(2):174-7.

21. Mark KE, Wald A, Magaret AS, Selke S, Olin L, Huang M-L, et al. Rapidly cleared episodes of herpes simplex virus reactivation in immunocompetent adults. Journal of Infectious Diseases. 2008;198(8):1141-9.

22. Awad SF, Sgaier SK, Tambatamba BC, Mohamoud YA, Lau FK, Reed JB, et al. Investigating Voluntary Medical Male Circumcision Program Efficiency Gains through Subpopulation Prioritization: Insights from Application to Zambia. PloS one. 2015;10(12):e0145729.

23. Weinstein M, Wood JW, Stoto MA, Greenfield DD. Components of age-specific fecundability. Population Studies. 1990;44(3):447-67.

24. Bernstein DI, Bellamy AR, Hook EW, Levin MJ, Wald A, Ewell MG, et al. Epidemiology, clinical presentation, and antibody response to primary infection with herpes simplex virus type 1 and type 2 in young women. Clinical infectious diseases. 2013;56(3):344-51.

25. Roberts CM, Pfister JR, Spear SJ. Increasing proportion of herpes simplex virus type 1 as a cause of genital herpes infection in college students. Sexually transmitted diseases. 2003;30(10):797-800.

26. Belshe RB, Leone PA, Bernstein DI, Wald A, Levin MJ, Stapleton JT, et al. Efficacy results of a trial of a herpes simplex vaccine. New England Journal of Medicine. 2012;366(1):34-43.

27. Kim HN, Meier A, Huang M-L, Kuntz S, Selke S, Celum C, et al. Oral herpes simplex virus type 2 reactivation in HIV-positive and-negative men. The Journal of infectious diseases. 2006;194(4):420-7.

28. Wald A. Sexual practices and oral and genital herpes simplex virus shedding patterns among a cohort with laboratory documented first episode genital HSV-1. The International Union against Sexually Transmitted Infections (IUSTI) World & European Congress, Dublin, Ireland. 2018.
